# Nonlinear Stability in a Free Boundary Model of Active Locomotion

**DOI:** 10.1007/s00205-025-02153-5

**Published:** 2025-12-10

**Authors:** Leonid Berlyand, C. Alex Safsten, Lev Truskinovsky

**Affiliations:** 1 Department of Mathematics and Huck Institute for Life Sciences, The Pennsylvania State University, University Park, USA; 2 Department of Mathematics, University of Maryland, College Park, USA; 3 ESPCI Paris, Paris, France

## Abstract

Contraction-driven self-propulsion of a large class of living cells can be modeled by a Keller-Segel system with free boundaries. The ensuing “active” system, exhibiting both dissipation and anti-dissipation, features stationary and traveling wave solutions. While the former represent static cells, the latter describe propagating pulses (solitary waves) mimicking the autonomous locomotion of the same cells. In this paper we provide the first proof of the asymptotic nonlinear stability of both of these solutions, static and dynamic. In the case of stationary solutions, the linear stability is established using the spectral theorem for compact, self-adjoint operators, and thus linear stability is determined classically, solely by eigenvalues. For traveling waves the picture is more complex because the linearized problem is non-self-adjoint, opening the possibility of a “dark” area in the phase space which is not “visible” in the purely eigenvalue/eigenvector approach. To establish linear stability in this case we employ spectral methods together with the Gearhart-Prüss-Greiner (GPG) theorem, which controls the entire spectrum via bounds on the resolvent operator. For both stationary and small-velocity traveling wave solutions, nonlinear stability is then proved for appropriate parameter values by showing that the nonlinear part of the problem is dominated by the linear part and then employing a Grönwall inequality argument. The developed novel methodology can prove useful also in other problems involving non-self-adjoint (non-Hermitian or non-reciprocal) operators which are ubiquitous in the modeling of “active” matter.

## Introduction

The ability of cells to self-propel is fundamental for many aspects of development, homeostasis, and disease, for instance, cells need to move to form tissues and their migration is also critical during tissue repair [31, 91, 93, 99]. The active machinery behind self-propulsion resides in the cytoskeleton—a meshwork of actin filaments with contractile cross-linkers represented by myosin motors. The main active processes in the cytoskeleton are the polymerization of actin fibers and the relative sliding of actin fibers induced by myosin motors [2]. The molecular and biochemical basis of these processes is basically known, however the corresponding mathematical theory is still under development and a variety of multiscale simulation approaches targeting various cell motility mechanisms can be found in the literature [8, 18, 20, 21, 47, 58, 70, 78, 98, 103].

Aiming at the development of a rigorous mathematical approach to stability analysis of such models, we focus in this paper on the simplest phenomenon of self-propulsion in a particular class of cells: keratocytes. They move by advancing the front through polymerization with a simultaneous formation of adhesion clusters. After the adhesion of the protruding part of the cell is secured, the cytoskeleton contracts due to activity of myosin motors. This contraction leads to detachment at the rear and depolymerization of the actin network. All three components of the motility mechanism (polymerization, contraction, and adhesion) depend upon continuous ATP hydrolysis and require intricate regulation by complex signaling pathways involving chemical and mechanical feedback loops [10, 95].

Contractile force generation is of fundamental importance for this mode of cell migration. Using actin fibers as a substrate, myosin motors [51] generate forces which are ultimately responsible for both the motility initiation and the steady locomotion of keratocytes [1, 24, 44, 68, 97]. In view of such central role of active contraction and to achieve relative analytical transparency of the mathematical analysis, we consider in this paper a prototypical model which emphasizes contraction as the main driving mechanism while accounting for polymerization and adhesion only in a schematic manner.

Our minimal model of cell motility is based on a one-dimensional projection of the complex intracellular dynamics onto the direction of motion. More specifically, we assume that the motor part of a cell can be viewed as a one-dimensional continuum with two free boundaries representing the front and the rear of the moving cell. We make a simplifying physical assumption that actin polymerization and de-polymerization can take place only on these boundaries and that these phenomena can be modeled as an influx of mass at the front boundary and its disappearance at the rear boundary. The adhesion is also treated in an over-simplified form as passive spatially inhomogeneous viscous friction. Instead, the actomyosin contraction, which is the main player, is represented by active spatially inhomogeneous prestress [53, 61].

As it was first shown in [79, 80], the mathematical model, which captures all these physical effects while being amenable to rigorous mathematical analysis, reduces to the one-dimensional Keller-Segel system with free boundaries. In contrast to the conventional chemotaxic Keller-Segel model [56], here the same set of equations emerges in a purely mechanical setting; see [16, 19, 33, 53, 61, 63, 82] for the earlier insights along the same lines. In Sect. 2, where we present for convenience a short derivation of this model, we also highlight its universality (minimality) by showing that it can be obtained starting from rather different physical assumptions.

It is important to mention that alternative free-boundary-type models of cell motility, emphasizing various other components of the self-propulsion machinery, have been used in numerical simulations [57, 71, 72, 83, 89] and, in some cases, also subjected to rigorous mathematical analysis [28, 29]. Closely related free boundary models describing tumor growth have been also studied both analytically and numerically [30, 45, 46, 49]. Our paper differs from all this mathematical work on free boundary modeling of locomotion in its emphasis on the non-self-adjoint property of the linearized operator resulting from both nonlocality [52] and activity [5]. Note that phase field models of cell motility, representing a mathematical proxy to our free boundary formulation (front capturing instead of front tracking [15, 32]), have been also a subject of extensive research efforts [12–14, 102, 104]. However, while the corresponding models allow for very efficient numerical simulations, they are usually not as readily amenable for rigorous stability analysis, and therefore will not be addressed in the present purely analytical study.

The one-dimensional Keller-Segel system with free boundaries is known to possess a family of pulse-like traveling wave solutions, which describe steady autonomous locomotion of individual cells [79, 80, 86, 87]. These solutions, which can be interpreted as solitary waves, bifurcate from a family of stationary (static) solutions, representing non-moving cells. The role of bifurcation parameter is played by a non-dimensional measure of the level of internal activity with both static and dynamic solutions being “active” in the sense that they consume and dissipate energy. In [79, 80] the whole variety of stationary solutions was constructed analytically and the nature of the corresponding static-dynamic bifurcation was determined using weakly nonlinear analysis involving a standard approach based on Lyapunov-Schmidt reduction [48]; significant numerical evidence that traveling waves bifurcating from homogeneous stationary states have finite reserve of stability was also obtained. In [86, 87], the same bifurcation between stationary and traveling wave solutions was studied in two dimensions, and the configurations of the traveling wave solutions were computed both analytically (close to the bifurcation point) and numerically (away from it). Linear stability was addressed for both stationary and traveling wave solutions with the eigenvalue-based stability condition computed explicitly.

### Summary of the Main Results

The present paper begins with a derivation of the free-boundary model of cell motion in Sect. 2 followed by nondimensionalization and a further derivation of the “stiff limit” of this model as the cell’s elastic stiffness (see (3)) becomes infinite in Sect. 3. The ultimate model on which the bulk of our analysis is focused is

1 $$\begin{aligned} {\left\{ \begin{array}{ll} \partial _t m=m''+\phi '(1/2)m'-(m\phi ')' &  -1/2<x<1/2\\ -Z\phi ''+\phi =Pm &  -1/2<x<1/2, \end{array}\right. } \end{aligned}$$

where *m* represents myosin density and satisfies Neumann boundary conditions and $\phi$ represents pressure and satisfies periodic boundary conditions (here, the prime $'$ denotes spatial derivative). The remaining parameters in the model are the magnitude of viscosity force (viscosity of the cytogel) in the cell denoted by *Z* and the magnitude of activity force (activity rate of the myosin) in the cell, denoted *P*.

In Sect. 4, we focus on homogeneous stationary solutions to the model. The main achievement of this section is Theorem 4.1, which can be summarized as follows:

**Nonlinear stability of stationary solutions:** The stationary solutions of (1) are nonlinearly exponentially stable when the physical parameters *P*, *Z* are in the range that is explicitly determined by a transcendental equation.

In Sect. 5, we examine traveling wave solutions to the model, showing in Theorem 5.5 that a family of traveling waves bifurcates from the stationary solution at $P=P_0$ via the Crandall-Rabinowitz (CR) theorem [27]. We describe this family of traveling wave solutions parameterized by their velocity *V* and the corresponding activity parameter $P_{TW}(V)$ which characterizes the total amount of activity of myosin motors required to move with velocity *V*.

Finally, Sect. 6 is focused on stability of traveling wave solutions, with the primary result of this paper being Theorem 6.1, can be which is summarized as follows:

**Nonlinear stability of traveling waves via non-self-adjoint spectral analysis:** There exists $Z^*,V^*>0$ so that if $Z>Z^*$, $|V|<V^*$ and $P=P_{TW}(V)$, then the traveling wave with velocity *V* is exponentially stable.

The key observation in the proof of this theorem is that the standard stability analysis based on eigenvalues and eigenvectors is not sufficient. Indeed, due to non-self-adjointness of the linearized operator, eigenvectors may not span the entire phase space and alternative techniques based on resolvent analysis were developed. The second challenge in this proof is the transition from linear to nonlinear stability. Evaluated at an arbitrary perturbation of the traveling wave solution, the nonlinear operator for this model can be written as its linearization about the traveling wave plus a nonlinear part bounded by the product of the $L^2$ and $H^1$ norms of the perturbation. We then show via a series of subtle bounds (reminiscent of parabolic regularization) that the $H^1$ norm of a solution to (1) can be controlled by the $L^2$ norm provided the $H^1$ norm is small at $t=0$. This allows us to show that the linear part of (1) dominates the nonlinear part in the vicinity of the traveling wave, allowing the linear stability to be used to prove nonlinear stability.

### Methods and Challenges

The main difficulty in the stability analysis of the traveling wave solutions resides in the non-self-adjoint (non-Hermitian, or non-reciprocal) nature of the corresponding linearized operator [35], which is an important general feature of PDE models of “active" matter [5, 34, 39, 92, 101]. It is known, for instance, that for non-self-adjoint (NSA) operators, eigenvectors do not necessarily span the entire domain of the operator. Therefore, common stability analysis, e.g. [3, 73], based only on eigenvalues and eigenvectors may not be sufficient [96].

We recall that when the linearized problem is self-adjoint, the eigenmodes of the stable system can be divided into stable (corresponding to eigenvalues with negative real part), and center (with zero real part eigenvalues). In the nonlinear setting, solutions in the corresponding stable manifold would then be controlled (bounded) by solutions in the center manifold. Furthermore, a nonlinear ODE can be derived for solutions in the center manifold, from which it can be shown that all such solutions asymptotically approach the equilibrium. It would then mean that all other solutions also approach it. The key assumption in this approach to stability is that eigenvectors of the linearized operator span the entire domain of the operator. This may not be the case for NSA operators which typically exhibit a “dark” area in the phase space which is not “visible” in the purely eigenvalue/eigenvector approach. We address this challenge using directly resolvent analysis instead of relying solely on eigenvalues.^1^

In the NSA case, where we have to deal with the entire spectrum of the linearized operator, linear stability can be established by applying the Gearhart-Prüss-Greiner (GPG) theorem [41] which operates directly with bounds on the resolvent of the linear operator. Specifically, when eigenvectors do not span the domain of the operator *A*, the GPG theorem turns to the analysis of another operator

2 $$\begin{aligned} R_\mu =(\mu I-A)^{-1} \end{aligned}$$

with the parameter $\mu$ having a positive real part. The crucial step is then to bound $R_\mu$ away from any point of the entire spectrum, not just the eigenvalues. In particular, even in infinite dimensions, such a bound rules out the cases when a sequence of eigenvalues has negative real parts converging to zero.

After the linear stability is established, a natural step in checking the nonlinear stability would be, at least in finite dimensions, to use the Hartman-Grobman (HG) theorem [4]. However, even in this case, this theorem requires the absence of eigenvalues with zero real part. Our problem has a zero eigenvalue (a slow manifold) which appears in the linearized operator due to translational symmetry. To overcome this complication, we use the notion of “stability up to shifts”, see for instance [87], and prove the appropriate analog of the HG theorem specifically tailored for our infinite dimensional problem. While there are several extensions of the HG theorem to infinite dimensions, e.g. [6], most of these results apply to a smooth nonlinear operator mapping a Banach space to itself whereas in our parabolic PDE problem, the operator maps a Sobolev space $H^2$ to $L^2$. The existing HG type results for parabolic equations [67] are also not directly applicable to our problem. Our original approach is based on establishing subtle bounds on the derivatives of the solution in the neighborhood of a pitchfork bifurcation which allow one to decide when the linear part of the nonlinear operator dominates its nonlinear part. Our result is then equivalent to establishing the existence of a Lyapunov function (or rather Lyapunov functional in our infinite dimensional setting) for the pulse-like traveling wave solutions with synchronously moving free boundaries, see [11, 69, 90] for related results. We emphasize that our approach is readily generalizable to other PDE models where the task is to prove exponential stability of an emerging nontrivial solution in the vicinity of a bifurcation point.

While our approach is original, it is important to mention that a large variety of other methods for establishing nonlinear stability of traveling waves have been explored in the literature, see the reviews in [55, 75, 88]. In particular, several studies deal specifically with spectral stability of traveling wave solutions by showing that the spectrum of the linearized operator consists only of points with negative real part [64, 65, 76]. Most of these studies use the method of Evans function, which is a convenient tool for separating the eigenvalues from the continuous spectrum ubiquitous in traveling wave problems defined in unbounded domains [7, 23, 42]. We do not use the Evans function based approach for two reasons. First, our traveling waves are compact and there is no continuous spectrum for our problem. Second, in our specific problem, we can circumvent the use of Evans function by resorting to a simpler approach to calculate the leading eigenvalue developed in [26]. Other studies of linear and nonlinear stability of traveling waves, which use spectral theory to obtain bounds on the semigroup generated by the linear operator and then showing that the nonlinear problem is dominated by the linearization, can be found in [23, 54, 59]. While we basically follow the same strategy, our main spectral theoretic tool, which is the GPG theorem, is different from all those used in the previous studies.

## The Model

In this Section we briefly explain how our one-dimensional Keller-Segel system with free boundaries can be derived from physical considerations. To emphasize that this model is both minimal and universal, we present two alternative derivations based on apparently contradicting assumptions that the material inside the cell is either infinitely compressible or infinitely incompressible.

In the original, infinitely compressible version of the model, proposed in [77, 79, 80], we start by writing the 1D force balance for a gel segment in the form

$$\begin{aligned} \sigma ' =\xi v,\end{aligned}$$

where $\sigma (x,t)$ is axial stress, *v*(*x*, *t*) is the velocity of the gel, $\xi$ is the coefficient of viscous friction. We denote a single spatial coordinate by *x* and time by *t*; prime denotes the spatial derivative. The assumption of infinite compressibility of the gel decouples the force balance equation from the mass balance equation. Specifically, by neglecting compressibility, we can write the constitutive relation for an active gel, representing the material inside the cell, in the form

$$\begin{aligned}\sigma =\eta v'+k m,\end{aligned}$$

where $\eta$ is the bulk viscosity, *m*(*x*, *t*) is the mass density of myosin motors and $k >0$ is a constant representing contractile pre-stress per unit motor mass. The density of motors is modeled by a standard advection–diffusion equation where the advection is perceived to be originating from the flow of actin [16, 50, 100] i.e.,

$$\begin{aligned} \partial _t m+ (m v)'-D m''=0, \end{aligned}$$

where *D* is the constant effective diffusion coefficient, see [80] for the discussion of its physical meaning. Behind this equation is the assumption that myosin motors, actively cross-linking the implied actin meshwork, are not only being advected by the network flow but can also diffuse due to the presence of thermal fluctuations. To ensure that the moving cell maintains its size, we follow [9, 38, 66, 77, 81] and introduce a phenomenological cortex/osmolarity mediated quasi-elastic non-local interaction linking the front and the back of the self-propelling fragment. More specifically, we assume that the boundaries of our moving active segment interact through an effective linear spring which regulates the value of the stress on the free boundaries $l_-(t)$ and $l_+(t)$:

3 $$\begin{aligned} \sigma _0^{\pm } =-k_e(L(t)-L_0)/L_0. \end{aligned}$$

Here $L(t)=l_{+}(t)-l_{-}(t)$ is the length of the moving segment, $k_e$ is the effective elastic stiffness and $L_0$ is the reference length of the spring. Finally, we assume that the free boundaries are not penetrable which means that they move with the internal flow. Therefore the following kinematic boundary condition must hold: $\partial _t{l}_{\pm }=v(l_{\pm })$. Our assumptions allow us to avoid addressing explicitly the mass balance equation since we effectively postulate that the addition of actin particles at the front is fully compensated by their synchronous removal at the rear, see [79, 80] for details. We also impose a zero flux condition for the active component $m'(l_{\pm }(t),t)=0$ ensuring that the average concentration of motors $m_0= L_0^{-1}\int _{l_-(t)}^{l_+(t)}m(x,t){\text {d}}x$ is preserved. To complete the setting of the ensuing mechanical problem we impose the initial conditions $l_{\pm }(0)=l_{\pm }^0$ and $m(x,0)=m^0(x).$ One can see that the resulting one-dimensional problem is equivalent to a dynamical system of a Keller–Segel-type with free boundaries, however, in contrast to the chemotaxic analog, the nonlocality here is mechanical rather than chemical in origin.

Next, we derive the same model under the incompressibility assumption as it was first proposed in [86, 87]. This alternative derivation emphasizes the paradigmatic nature of our problem. We also switch now to a more formal mathematical description as appropriate for the subsequent rigorous analysis.

The cell, whose cytoskeleton is now viewed as an incompressible fluid, is again modeled in a time-dependent interval centered at a point *c*(*t*) and with width *L*(*t*): $\Omega (t)=(c(t)-L(t)/2,c(t)+L(t)/2)\subset {\mathbb {R}}$. For each $t\ge 0$, the velocity of fluid in the cell is $u(x,t)\in {\mathbb {R}}$ for $x\in \Omega (t)$. Since the cell is thin in the dorsal direction, most of the fluid within the cell lies close to the cell membrane. Therefore, we may assume the flow of incompressible fluid is dominated by friction and follows Darcy’s law:

$$\begin{aligned} p' = \zeta u. \end{aligned}$$

Here, $p(\cdot ,t):\Omega (t)\rightarrow {\mathbb {R}}$ is the total pressure within the cell, $\zeta >0$ is the constant adhesion coefficient and the prime denotes spatial derivative; see Appendix D in [22] for the detailed physical derivation. Following [87], we write the equation for the total pressure in the form

$$\begin{aligned}p=\mu u'+km,\end{aligned}$$

where $m(\cdot ,t):\Omega (t)\rightarrow {\mathbb {R}}_+$ is the density of myosin within the cell, $\mu >0$ is the viscosity coefficient, and $k>0$ is the contractility of myosin. The incompressibility assumption suggests that the hydrostatic fluid pressure here should be viewed as a kinematic variable which in our 1D setting is constant and can be simply absorbed into *p*.

We further assume that, again, myosin density evolves in time according to the advection–diffusion equation, which, after applying Darcy’s law can be written in the form (see [87] for details):

4 $$\begin{aligned} \partial _t m = Dm''-(m\phi ')'. \end{aligned}$$

To ensure that the total myosin mass is conserved in time, the myosin density satisfies no-flux boundary conditions: $m'=0$.

A boundary condition for total pressure is obtained from an assumption that there is a non-constitutive global elastic restoring force due to osmotic effects or the cell membrane cortex tension. This non-local effect is modeled by the condition

5 $$\begin{aligned} p =-k_e(L(t)-L_0)/L_0, \end{aligned}$$

where $L_0$ is the length of the cell in a reference configuration, and $k_e>0$ is the elastic stiffness coefficient. Note that in view of the possibility for the fluid to escape in the dorsal direction, our 1D cell is effectively compressible despite the incompressibility of the fluid, so that the coefficient $k_e$ can be also interpreted as the inverse compressibility of the cell as a whole.

The ensuing 1D problem is analytically transparent due to the fact that the variable $\phi =p/\zeta$ satisfies a linear elliptic equation:

6 $$\begin{aligned} - \mu \phi '' + \zeta \phi = km. \end{aligned}$$

The complexity of the problem resides in the boundary conditions. Thus, as we have seen, the variable $\phi$ at the boundary satisfies the nonlocal condition of Dirichlet type. Furthermore, to find the time evolution of the interval $\Omega (t)$, we assume that both components of our binary mixture, solvent (actin) and solute (myosin), respect the kinematic (velocity-matching) boundary conditions of Hele-Shaw type:

7 $$\begin{aligned} \partial _tL(t)&=\phi '(c(t)+L(t)/2,t)-\phi '(c(t)-L(t)/2,t)\end{aligned}$$

8 $$\begin{aligned} \partial _tc(t)&=\frac{1}{2}\left( \phi '(c(t)+L(t)/2,t)+\phi '(c(t)-L(t)/2,t)\right) . \end{aligned}$$

While the resulting fluid model, introduced in [86, 87], is mathematically equivalent of the solid gel model introduced in [79, 80], it is the Hele-Shaw fluid formulation which opened the way towards two-dimensional analysis allowing one to track not only the position of a moving cell but also the evolution of its shape; the corresponding 2D version of the gel model can be found in [43].

## Infinite Rigidity Limit

Below we distinguish between two versions of our model of cell locomotion. The first one, to which we refer as “Model A”, assumes, as in the original derivation, that the size of the cell is controlled by an elastic spring, whose elastic modulus will be referred to as the “stiffness” parameter. By considering an asymptotic limit when such stiffness tends to infinity, we will derive a “stiff limit” of the original model to which we refer in what follows as “Model B.” In this limiting model, which turns out to be analytically much more transparent, the cell has a fixed size.

We recall that in the model A, the myosin density *m*, pressure $\phi$, length *L*, and center *c* satisfy the PDE system: 
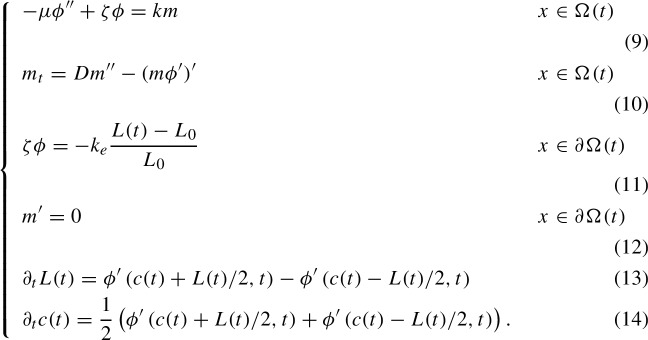
 An important feature of this model is that total myosin mass is conserved in time:

15 $$\begin{aligned} \frac{{\text {d}}}{{\text {d}}t}\int _{c(t)-L(t)/2}^{c(t)+L(t)/2}m(x,t)\,{\text {d}}x=0. \end{aligned}$$

To nondimensionalize this model, we rescale *x* by $x\rightarrow x/L_0$ and accordingly $L\rightarrow L/L_0$, $c\rightarrow c/L_0$. We then rescale time by $t\rightarrow tD/L_0^2$, pressure by $\phi \rightarrow \phi \zeta /k_e$ and myosin density by $m\rightarrow m L_0^2/M$ where *M* is the total myosin mass.

After such normalization, the variables *x*, *t*, *m*, $\phi$, *L*, and *c* are all dimensionless quantities and the PDE system (9)–(14) can be re-written in the form 
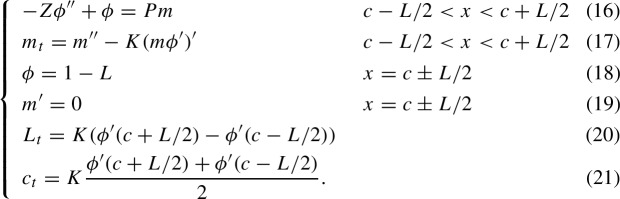
 Here we introduced the main non-dimensional parameters of the model

22 $$\begin{aligned} Z=\frac{\mu }{\zeta L_0^2}\quad P=\frac{kM}{k_eL_0}\quad K=\frac{k_e}{D\zeta }, \end{aligned}$$

representing dimensionless measures of bulk viscosity, activity level and the non-local stiffness, respectively. Note that in these rescaled coordinates, the total myosin mass is

23 $$\begin{aligned} \int _{c(t)-L(t)/2}^{c(t)+L(t)/2}m(x,t)\,{\text {d}}x=1. \end{aligned}$$

A simpler and analytically more tractable model can be obtained if we consider the limit when the “stiffness” coefficient $k_e$ tends to infinity, see also [80]. To this end, suppose $k_e=k_e^*/\varepsilon$ where $\varepsilon$ is a small, positive constant. Each of the coefficients *P*, *K* in (22) depend on $k_e$. Therefore, we denote them $P_\varepsilon =\varepsilon P_1$, and $K_\varepsilon =K_{-1}/\varepsilon$ respectively where

24 $$\begin{aligned} P_1=\frac{kM}{k_e^*L_0}\quad K_{-1}=\frac{k_e^*}{D\zeta }. \end{aligned}$$

For each $\varepsilon >0$, we now assume that $\phi _\varepsilon$, $m_\varepsilon$, $L_\varepsilon$, and $c_\varepsilon$ solve (16)–(21) with coefficients

$P_\varepsilon$, and $K_\varepsilon$ for $0\le t<T$ with initial conditions $m_\varepsilon (0,x)=\bar{m}(x)$, $L_\varepsilon (0)=\bar{L}$, and $c_\varepsilon (0)=\bar{c}$. We then expand each of $\phi _\varepsilon$, $m_\varepsilon$, $L_\varepsilon$, and $c_\varepsilon$ in small $\varepsilon$:

25 $$\begin{aligned} \phi _\epsilon&=\phi _0+\varepsilon \phi _1+O(\varepsilon ^2) \quad  &   m_\varepsilon =m_0+\varepsilon m_1+O(\varepsilon ^2)\end{aligned}$$

26 $$\begin{aligned} L_\varepsilon&=L_0+\varepsilon L_1+O(\varepsilon ^2) \quad  &   c_\varepsilon =c_0+\varepsilon c_1+O(\varepsilon ^2). \end{aligned}$$

The next step is to substitute these expansions along with the expansions for $P_\varepsilon$ and $K_\varepsilon$ into our equations and compare terms of like power in $\varepsilon$. Note that the free boundary requires that the boundary conditions be expanded not only in $\varepsilon$, but also *x* so that all boundary conditions are evaluated at $x=c_0\pm L_0/2$.

In zeroth order, (16) becomes $-Z\phi _{0,xx}+\phi _0=0$ with boundary condition $\phi _0=1-L_0$. We explicitly calculate that

27 $$\begin{aligned} \phi _0=(1-L_0)\frac{\cosh \left( \frac{c_0-x}{\sqrt{Z}}\right) }{\cosh \left( \frac{L_0}{2\sqrt{Z}}\right) .} \end{aligned}$$

In order $-1$, (17) has only one nontrivial term:

28 $$\begin{aligned} K_{-1}(m_0\phi _{0,x})'=0. \end{aligned}$$

We conclude that $m_0\phi _{0,x}$ is constant in *x*. Since the initial condition $\bar{m}$ is arbitrary in $H^2(-L_0/2,L_0/2)$, and since $\phi _0$ given by (27) does not depend on $m_0$, this can only be accomplished if $\phi _{0,x}=0$. This, in turn, implies $\phi _0=0$ and $L_0=1$.

Since $\phi _0=0$, in zeroth order, (17) becomes

29 $$\begin{aligned} m_{0,t}=m_{0,xx}-K_{-1}(m_0\phi _{1,x})'. \end{aligned}$$

Here $\phi _1$ satisfies the first order expansions of (16) and its boundary condition (18):

30 $$\begin{aligned} {\left\{ \begin{array}{ll} -Z\phi _{1,xx}+\phi _{1}=P_1m_0 &  c_0-L_0/2<x<c_0+L_0/2\\ \phi _1=-L_1 &  x=c_0\pm L_0/2 \end{array}\right. } \end{aligned}$$

In zeroth order, (20) becomes

31 $$\begin{aligned} L_{0,t}=K_{-1}(\phi _{1,x}(c_0+L_0/2)-\phi _{1,x}(c_0-L_0/2)). \end{aligned}$$

On the other hand, we know that $L_0\equiv 1$, so $L_{0,t}=0$. Therefore, $\phi _1$ has to satisfy three boundary conditions:

32 $$\begin{aligned}  &   \phi _1(c_0-L_0/2)=-L_1,\quad \phi _1(c_0+L_0/2)=-L_1,\quad \nonumber \\  &   \phi _{1,x}(c_0-L_0/2)=\phi _{1,x}(c_0+L_0/2). \end{aligned}$$

We conclude that $L_1$ is determined by these three conditions. Since $\phi _1$ satisfies periodic boundary conditions, we may determine $c_0$ by the zeroth order expansion of (21):

33 $$\begin{aligned} c_{0,t}=K_{-1}\phi _{1,x}(c_0+L_0/2). \end{aligned}$$

To complete the derivation of our “Model B”, we omit the subscript indices so we write $m=m_0$, $L=L_0=1$, $c=c_0$. It is also convenient to denote $\phi =K_{-1}\phi _1$ and introduce a new dimensionless parameter

$$\begin{aligned}P=P_1K_{-1}.\end{aligned}$$

We can then formulate the stiff limit of our model in the form of a reduced PDE system: 
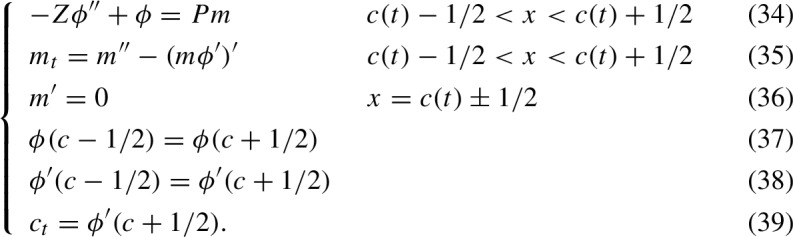
 Once again, the total myosin mass is conserved and its value is 1. Due to its relative simplicity, combined with the ability to still represent the main physical effects, model (34)-(39), first introduced formally in [80], will be the main focus of the present study.

Note first that Model B has stationary solution

$$\begin{aligned}m=1,\,\,\,\phi =P.\end{aligned}$$

As we are going to see, for *P* sufficiently small, such solution is exponentially stable, but for large *P*, it becomes unstable and bifurcates into traveling wave solutions. In the linearization of Model B about traveling waves, the invariance of traveling waves to translation manifests itself through a zero eigenvalue. By factorizing the shifts, the corresponding eigenvector is identified with 0, and in this way the zero eigenvalue is effectively removed.

The factorization of the shifts is accomplished by changing coordinates via the transformation $x\rightarrow x-c(t)$. In the new coordinates, (34)-(39) become 
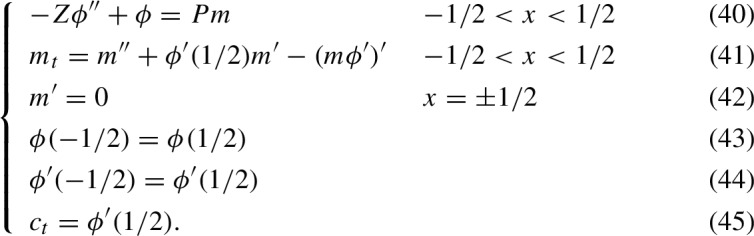
 Observe that the center coordinate *c* is partially decoupled from *m*. Therefore, we can drop the *c* coordinate, effectively grouping together all solutions that are the same up to a translation of the center in particular, a stationary solution to (40)–(44) becomes an element of an equivalence class of traveling wave solutions to (34)–(39). Furthermore, we see that if a stationary solution to (40)–(45) is exponentially stable, then the corresponding traveling wave solutions to (34)–(39) are also exponentially stable “up to shifts". Here we imply that a solution whose initial condition is a perturbation of a traveling wave solution may converge to a different traveling wave solution with the same velocity, but a different (“shifted”) center coordinate.

In what follows we refer to the result of transforming coordinates in Model B and dropping the *c* component as “Model C,” which we formulate more succinctly

46 $$\begin{aligned}  &   \partial _t m=F_C(m)=m''+\phi '(1/2)m'-(m\phi ')',\quad \nonumber \\  &   \qquad {\left\{ \begin{array}{ll} -Z\phi ''+\phi =Pm &  -1/2<x<1/2\\ \phi (1/2)=\phi (-1/2)\\ \phi '(-1/2)=\phi '(1/2).\end{array}\right. } \end{aligned}$$

The domain of the nonlinear operator $F_C$ is

$$X^2_C:=\left\{ m\in H^2(-1/2,1/2):m'(\pm 1/2)=0,\,\int _{-1/2}^{1/2}m\,{\text {d}}x=1\right\} .$$

## Nonlinear Stability of Stationary Solutions

We can now address the stability (up to shifts) of homogeneous stationary solutions $m_S:=1$ and $\phi =P$ of model B, by analyzing the $m_S=1$ solution to model C its; nonlinear exponential stability is captured in the following theorem:

### Theorem 4.1

Let $Z>0$. Then there exist $P_0>\pi ^2Z$ and $\varepsilon ,r>0$ such that if $0<P<P_0$ and $m_0\in H^1(-1/2,1/2)$ with $\Vert m_0-m_S\Vert _{H^1}<\varepsilon$, then for any solution *m*(*x*, *t*) to $\partial _t m=F_C(m)$ with $m(0,x)=m_0(x)$ we have

47 $$\begin{aligned} \Vert m(\cdot ,t)-m_{S}\Vert _{L^2}\le \Vert m_0-m_S\Vert _{L^2}e^{-rt}. \end{aligned}$$

### Remark 4.2

In Sect. 5, we will find $P_0=P_0(Z)$ as a solution to the explicit transcendental equation (96). Thus, Theorem 4.1 can be stated as a guarantee of exponential stability of $m_S$ provided the model parameters *P* and *Z* are in the set $\{(P,Z):0<Z\;\text {and}\;0<P<P_0(Z)\}$ where $P_0$ can be computed numerically.

A physical interpretation of Theorem 4.1 is that if the activity rate *P* is low enough relative to the viscosity *Z*, then the cell cannot move, and so stationary solutions in our model are stable. The proof of the nonlinear stability Theorem 4.1 can be broken down into two parts: (1) a proof of linear stability, and (2) a proof that close to the stationary solution, the nonlinear part of the model is dominated by the linear part. The former is largely a classical proof, though we include all details for completeness. The latter is a novel proof centered on the Gagliardo-Nirenberg theorem and the concept of diffusive regularization.

We begin the process of the proof of Theorem 4.1 with a study of linear stability. The linearization of model C is

48 $$\begin{aligned} S_Cu=DF_C(1)u=u''-\phi '', \end{aligned}$$

where $\phi$ is defined as in (46) (with $m=u$) and $u\in \tilde{X}^2_C:=\left\{ u\in H^2(-1/2,1/2):\right. \left. u'(\pm 1/2)=0,\,\int _{-1/2}^{1/2}u\,{\text {d}}x=0\right\}$. We first prove the following theorem establishing the linear stability of stationary states:

### Theorem 4.3

Let $Z>0$. Then there exist $P_0>\pi ^2Z$ and $\omega >0$ such that if $P<P_0$ and if *m*(*x*, *t*) solves $\partial _t u=S_Cu$ in $\tilde{X}_2$ with $u(0,x)=u_0(x)$, then

49 $$\begin{aligned} \Vert u(\cdot ,t)\Vert _{L^2}<\Vert u_0\Vert _{L^2}e^{-\omega t}. \end{aligned}$$

The proof of Theorem 4.3 is classical, and relies on the spectral theorem for compact self-adjoint operators [60], which states that a compact, self-adjoint operator has a basis of eigenvectors. The operator $S_C$ is not compact, but we will show that its inverse is, and $S_C$ shares the eigenvectors of its inverse. Therefore, we may reduce the problem of linear stability to the problem of stability of individual eigenstates, with the exponential decay in Theorem 4.3 given by a uniform negativity of the corresponding eigenvalues. To complete this proof, we must only show (via three Lemmas below) that (i) $S_C$ is self-adjoint, (ii) all eigenvalues of $S_C$ are negative and bounded away from zero, and (iii) $S_C$ has compact inverse. While the proofs of Theorem 4.3 and the supporting results Proposition 4.6 and Lemma 4.7 are classical, we include them for completeness.

First we prove self-adjointness. Let *X* be a Hilbert space and let $D(A)\subset X$ be a dense subspace of *X* which is the domain of an operator $A:D(A)\rightarrow X$. Recall that the adjoint of *A* is an operator $A^*$ such that $\langle u,A^*v\rangle =\langle Au,v\rangle$ for all $u,v\in D(A)$. The operator *A* is self-adjoint if $A=A^*$. Therefore, we introduce the following bilinear form to determine whether or not an operator is self-adjoint:

### Definition 4.4

Let *X* be an inner product space, and let $A:X\rightarrow X$. The *adjoint commutator* of *A* is $H:X\times X\rightarrow {\mathbb {R}}$ defined by

50 $$\begin{aligned} H(u,v)=\langle Au,v\rangle -\langle u,Av\rangle . \end{aligned}$$

If the adjoint commutator is identically zero, then *A* is self-adjoint. Otherwise, *A* is non-self-adjoint.

### Lemma 4.5

The linearization $S_C$ of $F_C$ about the stationary solution $m_S=1$ is self-adjoint with respect to the $L^2$ inner product.

### Proof

Let $H:\tilde{X}_2\times \tilde{X}_2\rightarrow {\mathbb {R}}$ be the adjoint commutator of $S_C$. Let $u_1,u_2\in \tilde{X}_2$. Let $\phi _i$ solve $-Z\phi _i''+\phi _i=Pu_i$ with periodic boundary conditions. Then

51 $$\begin{aligned} H(u_1,u_2)&=\int _{-1/2}^{1/2} u_2(u_1''-\phi _1'')\,{\text {d}}x-\int _{-1/2}^{1/2}u_1(u_2''-\phi _2'')\,{\text {d}}x \end{aligned}$$

52 $$\begin{aligned}&=\int _{-1/2}^{1/2}(u_1'u_2'-u_2'u_1')\,{\text {d}}x+\int _{-1/2}^{1/2}\left[ u_1\frac{\phi _2-Pu_2}{Z}-u_2\frac{\phi _1-Pu_1}{Z}\right] \,{\text {d}}x\end{aligned}$$

53 $$\begin{aligned}&=\frac{1}{PZ}\int _{-1/2}^{1/2}\left[ (-Z\phi _1''+\phi _1)\phi _2-(-Z\phi _2''+\phi _2)\phi _1\right] \,{\text {d}}x\end{aligned}$$

54 $$\begin{aligned}&=\frac{1}{P}\int _{-1/2}^{1/2}(\phi _1'\phi _2'-\phi _2'\phi _1')\,{\text {d}}x\end{aligned}$$

55 $$\begin{aligned}&=0. \end{aligned}$$

$$\square $$

Now we show the negativity of the eigenvalues of $S_C$.

### Proposition 4.6

If $P/Z<\pi ^2$, then all eigenvalues of $S_C$ are negative and bounded away from 0.

### Proof

Assume $P/Z<\pi ^2$. Let *u* be an eigenvector of $S_C$ and let $\lambda$ be its eigenvalue. Since $S_C$ is self adjoint, $\lambda$ is real and *u* is real-valued. Without loss of generality, we may assume $\Vert u\Vert _{L^2}=1$. Let $\phi$ solve $-Z\phi ''+\phi =Pu$ with periodic boundary conditions on $(-1/2,1/2)$. Then *u* and $\lambda$ satisfy $u''-\phi ''=\lambda u$. Multiplying by *u* and integrating we find that

$$\begin{aligned} \lambda&=\int _{-1/2}^{1/2}u''u-\phi ''u\\&=-\int _{-1/2}^{1/2}u'^2\,{\text {d}}x-\frac{1}{Z}\int _{-1/2}^{1/2}(\phi -Pu)u\,{\text {d}}x\\&=-\int _{-1/2}^{1/2}u'^2\,{\text {d}}x+\frac{P}{Z}\int _{-1/2}^{1/2} u^2\,{\text {d}}x-\frac{1}{PZ}\int _{-1/2}^{1/2}\phi (-Z\phi ''+\phi )\,{\text {d}}x\\&=-\int _{-1/2}^{1/2}u'^2\,{\text {d}}x+\frac{P}{Z}-\frac{1}{P}\int _{-1/2}^{1/2}\phi '^2\,{\text {d}}x-\frac{1}{PZ}\int _{-1/2}^{1/2}\phi ^2\,{\text {d}}x\\&\le -\int _{-1/2}^{1/2}u'^2\,{\text {d}}x+\frac{P}{Z}. \end{aligned}$$

It is well known that the optimal constant in the Poincaré inequality in an interval of length 1 is $1/\pi$ [74]. The Poincaré inequality applies to *u* since $\int _{-1/2}^{1/2}u(x)\,{\text {d}}x=0$. Therefore, we conclude that

56 $$\begin{aligned} \lambda \le \frac{P}{Z}-\pi ^2<0. \end{aligned}$$

Therefore all eigenvalues of $S_C$ are negative and bounded away from 0. $\square$

While Proposition 4.6 gives the sufficient condition $P/Z<\pi ^2$ for the negativity of the eigenvalues of $S_C$, it is not optimal in the sense that the eigenvalues of $S_C$ may be negative for *P*/*Z* larger than $\pi ^2$. However, for fixed *Z* and sufficiently large *P*, there are positive eigenvalues of $S_C$. To see this, observe that $u(x)=\cos (2\pi x)$ is an eigenvector of $S_C$ (since it is also an eigenvector of $-Z\phi ''+\phi$ with periodic boundary conditions on $(-1/2,1/2)$). Its eigenvalue is $-4\pi ^2+\frac{P}{Z}\left( 1+\frac{1}{4\pi ^2Z}\right) ^{-1}$ (note however that this is not the largest eigenvalue). If, for fixed $Z>0$, the parameter *P* is large enough, this eigenvalue is positive. This observation hints that for some critical value $P_0>\pi ^2 Z$ of *P*, the largest eigenvalue of $S_C$ will reach zero, and for $P>P_0$. For given *Z*, we define

57 $$\begin{aligned} P_0= &   \sup \{\hat{P}>0:\text {all eigenvalues of }S_C\nonumber \\  &   \qquad \text { are negative and bounded away from }0\text { for }P<\hat{P}\} \end{aligned}$$

or equivalently,

58 $$\begin{aligned} P_0=\inf \{P>0:\text {the largest eigenvalue of }S_C\text { is zero}\}. \end{aligned}$$

**Fig. 1:**
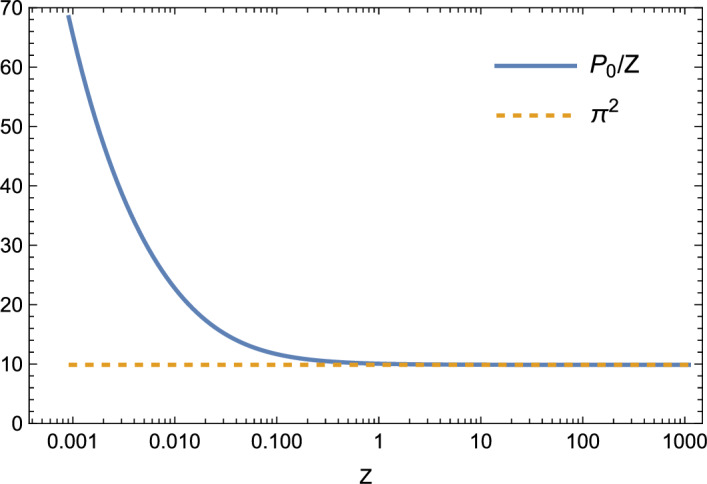
The numerically calculated value of $P_0/Z$ compared to $\pi ^2$ for a wide range of *Z* values

We will examine properties of $P_0$ in Sect. 5. In particular, in Theorem 5.5, we will see that the value of $P_0$ is the smallest positive, nontrivial solution to (96) below. In Lemma 5.8, we will see that for large *Z*, $P_0\approx \pi ^2 Z$. In fact, Fig. 1 shows that Proposition 4.6 provides an approximation of $P_0/Z$ that is close to optimal for all positive *Z* other than $Z\ll 1$.

Now we show that the inverse of the linearization $S_C$ is compact.

### Lemma 4.7

Given $Z>0$, if $P<P_0$, then $S_C$ is invertible and $S_C^{-1}:X\rightarrow X$ is compact.

### Proof

Assume $P<P_0$. Then 0 is not an eigenvalue of $S_C$. That $S_C$ is invertible follows from the Lax-Milgram Theorem (see Proposition 6.4 for details).

First we show that $S_C^{-1}$ is bounded. Suppose, to the contrary that it is unbounded. Then there exist sequences $(v_k)\subset \tilde{X}^2_C$ and $(w_k)\subset L^2(-1/2,1/2)$ such that

59 $$\begin{aligned} S_Cv_k=w_k,\quad \Vert v_k\Vert _{L^2}=1,\quad \Vert w_k\Vert _{L^2}\le 1/k. \end{aligned}$$

Let $\phi _k$ satisfy $-Z\phi _k''+\phi _k=Pv_k$ with periodic boundary conditions. Then the following sequence is bounded:

60 $$\begin{aligned} \begin{aligned} \langle w_k,v_k\rangle _{L^2}&=\langle S_Cv_k,v_k\rangle _{L^2}=-\Vert v_k'\Vert _{L^2}^2+\langle \phi _k'',v_k\rangle _{L^2}. \end{aligned} \end{aligned}$$

The sequence $\langle \phi _k'',v_k\rangle _{L^2}$ is bounded in *k* due to Proposition 7.1 in Appendix A. Since the (60) as a whole is bounded, we conclude that $\Vert v_k'\Vert _{L_2}$ is bounded in *k* as well.

Since $\Vert v_k\Vert _{L^2}$ and $\Vert v_k'\Vert _{L^2}$ are both bounded, we conclude that $(v_k)$ is bounded with respect to the $H^1$ norm. By the Banach-Alaoglu Theorem [84], there is a subsequence also called $v_k$ which converges weakly in $H^1(-1/2,1/2)$ and thus also in $L^2$. By Morrey’s inequality [37] and the Arzela-Ascoli theorem [17], $H^1(-1/2,1/2)\subset \subset L^2(-1/2,1/2)$, so we may assume that $(v_k)$ converges strongly to some $v\in L^2$. Since $\Vert w_k\Vert _{L^2}\le 1/k$, $w_k\rightarrow 0$ in $L^2(-1/2,1/2)$. Therefore, *v* is a weak solution to $S_Cv=0$. Since $\lambda I-S_C$ is invertible, we conclude that $v=0$. However, since $\Vert v_k\Vert _{L^2}=1$, we also have $\Vert v\Vert _{L^2}=1$, a contradiction. Therefore, $S_C^{-1}$ is a bounded operator.

Now we show that $S_C^{-1}:L^2(-1/2,1/2)\rightarrow L^2(-1/2,1/2)$ is compact. To that end, suppose that $(v_k)\subset \tilde{X}^2_C$ and $(w_k)\subset L^2(-1/2,1/2)$ such that

61 $$\begin{aligned} S_Cv_k=w_k\quad \text {and}\quad \Vert w_k\Vert _{L^2}\le 1. \end{aligned}$$

We need to show that there $(v_k)$ has a convergent subsequence. But this follows from the same logic as the above step. Since $S_C^{-1}$ is a bounded operator, $(v_k)$ is a bounded sequence in $L^2(-1/2,1/2)$. Therefore, once again each term in (60) is bounded, so $(v_k)$ has a weakly convergent subsequence in $X^1$ and a strongly convergent subsequence in $X^0$. Therefore, $S_C^{-1}$ is compact. $\square$

We can now prove the linear stability of stationary states.

### Proof of Theorem 4.3

Fix $Z>0$ and let $P_0$ be as described above so that if $P<P_0$, all eigenvalues of $S_C$ are negative and bounded away from 0. Then there exists $\omega >0$ so that for all eigenvalues $\lambda$ of $S_C$, $\lambda \le -\omega$. By Lemma 4.7, $S_C$ has compact inverse. By Lemma 4.5, $S_C$ is self-adjoint, and therefore $S_C^{-1}$ is also self-adjoint. By the spectral theorem [60], the eigenvectors of $S_C^{-1}$ form an orthogonormal set that spans a dense subset of $\tilde{X}_2$. Denote these eigenvectors $(v_n)$ for $n\in {\mathbb {N}}$ (since $\tilde{X}^2_C$ has countable dimension, we can enumerate the eigenvectors in this way). The eigenvectors of $S_C^{-1}$ are also eigenvectors of $S_C$. For each $n\in {\mathbb {N}}$, let $\lambda _n$ be the eigenvalue of $\lambda _n$ corresponding to $v_n$.

Suppose *u*(*x*, *t*) solves $\partial _t u=S_C u$ in $\tilde{X}_2$ with $u(0,x)=u_0(x)\in \tilde{X}^2_C$. Since the span of $(v_n)$ is dense in $\tilde{X}^2_C$, we may write any *u*(*x*, *t*) as an infinite linear combination of the eigenvectors

62 $$\begin{aligned} u(x,t)=\sum _{n=1}^\infty c_n(t)v_n(x) \end{aligned}$$

for coefficients $c_n:[0,\infty )\rightarrow {\mathbb {R}}$. Substituting this expansion into the linear evolution equation, we obtain

63 $$\begin{aligned} \sum _{n=1}^\infty c_n'(t)v_n(x)=\sum _{n=1}^\infty c_n(t)S_C v_n(x)=\sum _{n=1}^\infty \lambda _nc_n(t)v_n(x). \end{aligned}$$

By the orthogonality of the eigenvectors, we conclude that the sums must agree term-by-term, so for each *n*, $c_n'=\lambda _n c_n$, so

64 $$\begin{aligned} c_n(t)=c_n(0)e^{\lambda _n t}. \end{aligned}$$

Thus,

$$\begin{aligned} \Vert u(\cdot ,t)\Vert _{L^2}&=\sqrt{\langle u,u\rangle _{L^2}}=\sqrt{\sum _{n=1}^\infty c_n^2(t)}=\sqrt{\sum _{n=1}^\infty c_n^2(0)e^{2\lambda _nt}}\\&\quad =e^{-\omega t}\sqrt{\sum _{n=1}^\infty c_n^2(0)}=e^{-\omega t}\Vert u(0,\cdot )\Vert _{L^2}. \end{aligned}$$

Thus, the desired result holds. $\square$

It remains to show that the full, nonlinear stability result of Theorem 4.1 holds. To this end, we consider the *nonlinear part* $\Psi$ of $F_C$ defined for $u\in \tilde{X}^2_C$ by

65 $$\begin{aligned} \Psi (u)=F_C(1+u)-S_C u=\phi '(1/2)u'-(u\phi ')', \end{aligned}$$

where $\phi$ solves $-Z\phi ''+\phi =Pu$ with periodic boundary conditions in $(-1/2,1/2)$. The key to our proof of nonlinear stability is showing that

the nonlinear part $\Psi$ dominates the linear part $S_C$. We begin with a Lemma which gives a bound on $\Psi (u)$ in terms of *u*.

### Lemma 4.8

Let $\Psi :H^2([-1/2,1/2])\rightarrow H^1([-1/2,1/2])$ be defined as in (65). Then there exists $C>0$ independent of *u*, *P*, and *Z* such that

66 $$\begin{aligned} \Vert \Psi (u)\Vert _{L^2}\le \frac{CP}{Z}\Vert u\Vert _{L^2}\Vert u\Vert _{H^1}. \end{aligned}$$

### Proof

We make a direct calculation using estimates from Proposition 7.1 in Appendix A:

67 $$\begin{aligned} \Vert \Psi (u)\Vert _{L^2}^2&=\int _{-1/2}^{1/2}[(\phi '(1/2)-\phi '(x))u'(x)-u(x)\phi ''(x)]^2\,{\text {d}}x \end{aligned}$$

68 $$\begin{aligned}&\le 2\int _{-1/2}^{1/2}(\phi '(1/2)-\phi '(x))^2(u'(x))^2\,{\text {d}}x+2\int _{-1/2}^{1/2}(\phi ''(x))^2(u(x))^2\,{\text {d}}x\end{aligned}$$

69 $$\begin{aligned}&\le 4|\phi '(1/2)|^2\Vert u'\Vert _{L^2}^2+4\Vert \phi '^2u'^2\Vert _{L^1}+2\Vert \phi ''^2u^2\Vert _{L^1}\end{aligned}$$

70 $$\begin{aligned}&\le \frac{P^2}{Z^2}\Vert u\Vert _{L^1}^2\Vert u'\Vert _{L^2}^2+4\Vert \phi '\Vert _{L^\infty }^2\Vert u'\Vert _{L^2}^2+2\Vert \phi ''\Vert _{L^4}^2\Vert u\Vert _{L^4}^2\end{aligned}$$

71 $$\begin{aligned}&\le \frac{2P^2}{Z^2}\Vert u\Vert _{L^1}^2\Vert u\Vert _{H^1}^2+\frac{8P^2}{Z^2}\Vert u\Vert _{L^4}^4. \end{aligned}$$

By Hölder’s inequality, $\Vert u\Vert _{L^1}\le \Vert u\Vert _{L^2}$. From the Gagliardo-Nirenberg inequality, there exists $C_1>0$ independent of *u* such that

72 $$\begin{aligned} \Vert u\Vert _{L^4}\le C_1\Vert u\Vert _{H^1}^{1/2}\Vert u\Vert _{L^1}^{1/2}. \end{aligned}$$

Substituting (72) into (71) and letting $C^2=2+8C_1$, we obtain

73 $$\begin{aligned} \Vert \Psi (u)\Vert _{L^2}\le C\frac{P}{Z}\Vert u\Vert _{L^2}\Vert u\Vert _{H^1}. \end{aligned}$$

$$\square $$

Our next goal is to show that if $\Vert u\Vert _{L^2}$ is small, then so is $\Vert \Psi (u)\Vert _{L^2}$. However, Lemma 4.8 is not sufficient to accomplish this because even if $\Vert u\Vert _{L^2}$ is small, $\Vert u\Vert _{H^1}$ may be large. Therefore, the following lemma shows that if $\Vert u(\cdot ,t)\Vert _{L^2}$ is small for all *t*, then $\Vert u'(\cdot ,t)\Vert _{L^2}$ does not exceed $\Vert u'(\cdot ,0)\Vert _{L^2}$.

### Lemma 4.9

Let $Z>0$. Suppose $P<P_0$. Let $T,\varepsilon >0$ and let *u* be a solution to $\partial _tu=S_Cu+\Psi (u)$ in $C^1([0,T];\tilde{X}^2_C)$. There exists $U^*>0$ such that if $\Vert u'(\cdot ,0)\Vert _{L^2}<\varepsilon$ and $\Vert u(\cdot ,t)\Vert _{L^2}<U^*$ for all $0\le t\le T$, then

74 $$\begin{aligned} \Vert u'(\cdot ,t)\Vert _{L^2}\le \varepsilon \quad \text {for all }0\le t\le T. \end{aligned}$$

### Proof

Write the evolution equation for *u* as

75 $$\begin{aligned} \partial _t u-u''=-\phi ''+\Psi (u). \end{aligned}$$

Square both sides and integrate to obtain

76 $$\begin{aligned} \Vert -\phi ''+\Psi (u)\Vert _{L^2}^2&=\int _{-1/2}^{1/2} (\partial _t u)^2-2(\partial _t u)u''+(u'')^2\,{\text {d}}x\end{aligned}$$

77 $$\begin{aligned}&=\Vert \partial _t u\Vert _{L^2}^2+2\int _{-1/2}^{1/2}(\partial _t u')u'\,{\text {d}}x+\Vert u''\Vert _{L^2}^2\end{aligned}$$

78 $$\begin{aligned}&=\Vert \partial _t u\Vert _{L^2}^2+\frac{\text {d}}{{\text {d}}t}\Vert u'\Vert _{L^2}^2+\Vert u''\Vert _{L^2}^2. \end{aligned}$$

Thus,

79 $$\begin{aligned} \frac{\text {d}}{{\text {d}}t}\Vert u'\Vert ^2_{L^2}&\le \Vert -\phi ''+\Psi (u)\Vert _{L^2}^2-\Vert u''\Vert _{L^2}^2\end{aligned}$$

80 $$\begin{aligned}&\le 2\Vert \phi ''\Vert _{L^2}^2+2\Vert \Psi (u)\Vert _{L^2}^2-\Vert u''\Vert _{L^2}^2. \end{aligned}$$

From Lemma 4.8, there exists $C_1$ independent of *u* such that $\Vert \Psi (u)\Vert _{L^2}\le C_1\Vert u\Vert _{L^2}\Vert u\Vert _{H^1}.$ Moreover, by Proposition 7.1 in Appendix A, $\Vert \phi ''\Vert _{L^2}\le 2P/Z\Vert u\Vert _{L^2}$. Since $\int _{-1/2}^{1/2}u\,{\text {d}}x=0$ and $u'(\pm 1/2,t)=0$, we may apply the Poincaré inequality to both *u* and $u'$ with the optimal Poincaré constant $1/\pi$:

81 $$\begin{aligned} \Vert u\Vert _{L^2}\le \frac{1}{\pi }\Vert u'\Vert _{L^2}\quad \text {and}\quad \Vert u'\Vert _{L^2}\le \frac{1}{\pi }\Vert u''\Vert _{L^2}. \end{aligned}$$

Thus,

82 $$\begin{aligned} \frac{\text {d}}{{\text {d}}t}\Vert u'\Vert ^2_{L^2}&\le 8\frac{P^2}{Z^2}\Vert u\Vert _{L^2}^2+2C_1^2\Vert u\Vert _{L^2}^2\Vert u\Vert _{H^1}^2-\pi ^2\Vert u'\Vert _{L^2}^2\end{aligned}$$

83 $$\begin{aligned}&\le 8\frac{P^2}{Z^2}\Vert u\Vert _{L^2}^2+2C_1^2\Vert u\Vert _{L^2}^2(\Vert u\Vert _{L^2}+\Vert u'\Vert _{L^2})^2-\pi ^2\Vert u'\Vert _{L^2}^2\end{aligned}$$

84 $$\begin{aligned}&\le -\left( \pi ^2-4C_1^2\Vert u\Vert _{L^2}^2\right) \Vert u'\Vert _{L^2}^2+8\frac{P^2}{Z^2}\Vert w\Vert _{L^2}^2. \end{aligned}$$

Let $U^*<\pi /(4C_1)$. Then if $\Vert u\Vert _{L^2}\le U^*$ for all $0<t<T$,

85 $$\begin{aligned} \frac{\text {d}}{{\text {d}}t}\Vert u'\Vert ^2_{L^2}\le -R_1\Vert u'\Vert _{L^2}^2+R_2 \end{aligned}$$

where

86 $$\begin{aligned} R_1=\pi ^2-4C_1^2(U^*)^2>\frac{\pi ^2}{2}\quad \text {and}\quad R_2=8\frac{P^2}{Z^2}(U^*)^2. \end{aligned}$$

Let $q(t)=\Vert u'(\cdot ,t)\Vert _{L^2}^2-R_2/R_1$. Then *q* satisfies $q'\le -R_1q.$ By the Grönwall’s inequality, $q(t)\le q(0)e^{-R_1t}.$ We conclude that if $q(0)<0$, then $q(t)<0$ for all $t>0$. Thus, if $\Vert u\Vert _{L^2}\le U^*$, and if

87 $$\begin{aligned} \Vert u'(\cdot ,0)\Vert _{L^2}<\sqrt{\frac{R_2}{R_1}},\quad \text {then}\quad \Vert u'(\cdot ,t)\Vert _{L^2}<\sqrt{\frac{R_2}{R_1}} \end{aligned}$$

for all $t>0$. Letting $U^*<\varepsilon /(4\pi )$, we have

88 $$\begin{aligned} \sqrt{\frac{R_2}{R_1}}<4\pi U^*<\varepsilon , \end{aligned}$$

so the desired result holds. $\square$

### Proof of Theorem 4.1

Let $u=m-m_S=m-1$. Then *u* solves

89 $$\begin{aligned} {\left\{ \begin{array}{ll} \partial _t u=S_Cu+\Psi (u) &  -1/2<x<1/2,\;t>0\\ u'=0 &  x=\pm 1/2,\;t>0\\ u(\cdot ,0)=m_0-1:=u_0 &  t=0. \end{array}\right. } \end{aligned}$$

Let *S*(*t*) be the semigroup generated by $S_C$. By Theorem 4.3, there exists $\omega >0$ such that $\Vert S(t)\Vert _{L^2}\le e^{-\omega t}$. Applying Duhamel’s principle,

90 $$\begin{aligned} u(\cdot ,t)=S(t)u_0+\int _0^t S(t-\tau )\Psi (u(\tau ,\cdot ))\,d\tau . \end{aligned}$$

Taking the $L^2$ norm of both sides, we find that

91 $$\begin{aligned} \Vert u(\cdot ,t)\Vert _{L^2}\le e^{-\omega t}\Vert u_0\Vert _{L^2}+\int _0^t e^{\omega (\tau -t)}\Vert \Psi (u(\tau ,\cdot ))\Vert _{L^2}\,{\text {d}}\tau . \end{aligned}$$

Lemma 4.8 provides as estimate for $\Psi$ in terms of a constant *C*, leading to

92 $$\begin{aligned} \Vert u(\cdot ,t)\Vert _{L^2}\le e^{-\sigma t}\Vert u_0\Vert _{L^2}+ C\int _0^t e^{\omega (\tau -t)}\Vert u(\tau ,\cdot )\Vert _{L^2}\Vert u(\tau ,\cdot )\Vert _{H^1}\,{\text {d}}\tau . \end{aligned}$$

Let $\varepsilon =\omega \pi /(2 C(1+\pi ))$, and let $U^*$ be as in Lemma 4.9. Suppose that $\Vert u'(0,\cdot )\Vert _{L^2}<\varepsilon$ and $\Vert u(0,\cdot )\Vert _{L^2}<U^*$. Let

93 $$\begin{aligned} W=\{t\ge 0: \Vert u(\tau ,\cdot )\Vert _{L^2}\le U^*\text { for all }0\le \tau \le t\}. \end{aligned}$$

By continuity, *W* is a closed interval and $0\in W$. Thus, either $W=[0,\infty )$ or *W* has a positive maximum. Let $T\in W$. Then, after applying the Poincaré inequality and Lemma 4.9, for any $0\le t\le T$,

94 $$\begin{aligned} \begin{aligned} \Vert u(\cdot ,t)\Vert _{L^2}&\le e^{-\omega t}\Vert u_0\Vert _{L^2}+C\int _0^t e^{\omega (\tau -t)}\Vert u(\tau ,\cdot )\Vert _{L^2}\left( 1+\frac{1}{\pi }\right) \Vert u'(\tau ,\cdot )\Vert _{L^2}\,{\text {d}}\tau \\&\le e^{-\omega t}\Vert u_0\Vert _{L^2}+C\int _0^t e^{\omega (\tau -t)}\Vert u(\tau ,\cdot )\Vert _{L^2}\left( 1+\frac{1}{\pi }\right) \varepsilon \,{\text {d}}\tau \\&\le e^{-\omega t}\Vert u_0\Vert _{L^2}+\frac{\omega }{2}\int _0^t e^{\omega (\tau -t)}\Vert u(\tau ,\cdot )\Vert _{L^2}\,{\text {d}}\tau . \end{aligned} \end{aligned}$$

Therefore, by Grönwall’s inequality,

95 $$\begin{aligned} \Vert u(\cdot ,t)\Vert _{L^2}\le \Vert u_0\Vert _{L^2}e^{-\omega t/2} \end{aligned}$$

for all $0\le t\le T$. Therefore, $\Vert u(\cdot ,T)\Vert _{L^2}< U^*$ and so by continuity, $T\ne \max W$. Since $T\in W$ is arbitrary, we conclude that *W* does not have a maximum so $W=[0,\infty )$ and $\Vert u(\cdot ,t)\Vert _{L^2}\le U^*$ for all $t>0$. That is, (95) holds for all $t\ge 0$ provided $\Vert u(0,\cdot )\Vert _{L^2}<U^*$ and $\Vert u'(0,\cdot )\Vert _{L^2}<\varepsilon$. Note that from the proof of Lemma 4.9, $U^*<\varepsilon$. Therefore, the desired result holds. $\square$

## Traveling Waves

**Fig. 2:**
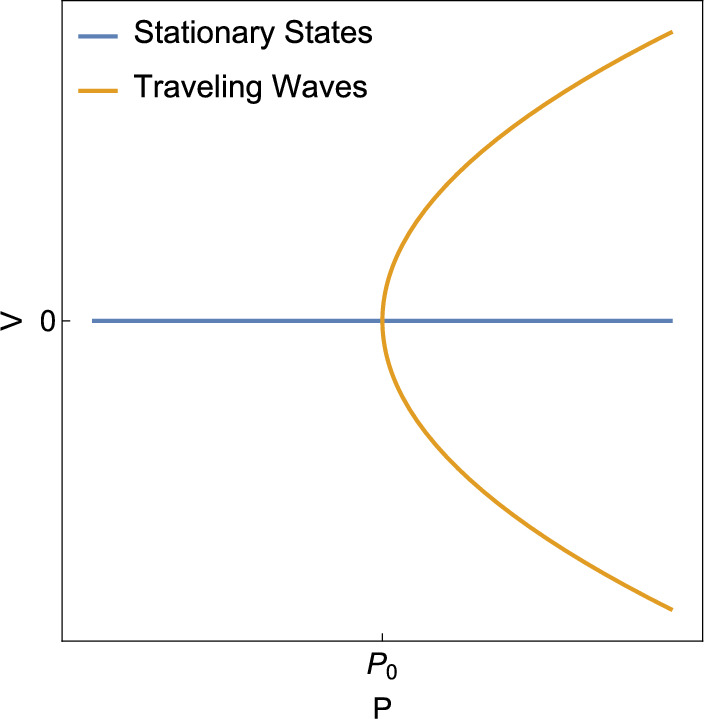
This diagram shows the pitchfork bifurcation from stationary states to traveling waves which is structurally the same in all three models

In this section, we show that for any $Z>0$, there exists a number $V^*>0$ and a smooth function $P_{TW}:(-V^*,V^*)\rightarrow {\mathbb {R}}$ such that if $P=P_{TW}(V)$, then there exists a traveling wave solution to the model B with velocity *V* and center $c=Vt$. This family of traveling wave solutions parameterized by *V* bifurcates from the family of stationary states at $V=0$ and $P=P_{TW}(0)=P_0$. As we show, this bifurcation is of the type illustrated in Fig. 2.

We will see that, for a given *Z*, the bifurcation occurs at a positive solution $P_0$ to

96 $$\begin{aligned}  &   \tanh \left( \frac{\sqrt{1-P_0}}{2\sqrt{Z}}\right) =P_0\frac{\sqrt{1-P_0}}{2\sqrt{Z}}\quad \text {or equivalently}\quad \nonumber \\  &   \tan \left( \frac{\sqrt{P_0-1}}{2\sqrt{Z}}\right) =P_0\frac{\sqrt{P_0-1}}{2\sqrt{Z}}. \end{aligned}$$

We write (96) in two forms to emphasize that $P_0$ be greater than or less than 1. Of course $P_0=1$ is a trivial solution, but we shall see that this solution does not correspond to a bifurcation, so we are interested in nontrivial solutions to (96). In fact, for large enough *Z*, there are infinitely many nontrivial positive solutions to (96), indicating that there are infinitely many bifurcations and infinitely many families of traveling waves. However, only one of these families of traveling waves is exponentially stable in small velocity, and this family corresponds to the smallest positive nontrivial solution to (96), and so when we write $P_0$, we refer to this solution. In the proof of Lemma 5.8, we see that if $Z<1/12$, then $P_0<1$ and if $Z>1/12$, then $P_0>1$. If $Z=1/12$, then $P_0=1$ is a degenerate root of (96) and the transversality condition in the Crandall-Rabinowitz Theorem used in Theorem 5.5 to prove the existence of bifurcation is not satisfied. Future study will be required for the $Z=1/12$ case.

### Remark 5.1

We note that the presence of the implied bifurcation was first discovered in [79, 80] where the structure of the associated bifurcation for both model A and model B was identified as well by using formal expansions. Here, in the framework of model B, we complement this earlier study not only by a rigorous analysis of the existence of the traveling wave solutions but also by providing a global analysis of the corresponding bifurcation.

Observe first that *m*(*x*, *t*) is a traveling wave solution with velocity *V* in model B if $m(x,t)=m_{TW}(x-Vt)$ where $m_{TW}$ satisfies

97 $$\begin{aligned}  &   {\left\{ \begin{array}{ll} m_{TW}''+Vm_{TW}'-(m_{TW}\phi _{TW}')'=0 &  -1/2<x<1/2\\ m_{TW}'(\pm 1/2)=0 &  \end{array}\right. } \quad \text {with}\nonumber \\  &   {\left\{ \begin{array}{ll} -Z\phi _{TW}''+\phi _{TW}=P(V)m_{TW} &  -1/2<x<1/2\\ \phi _{TW}(1/2)=\phi _{TW}(-1/2) &  \\ \phi _{TW}'(1/2)=\phi _{TW}'(-1/2)=V. &  \end{array}\right. } \end{aligned}$$

We further observe that a solution to this equation is $m_{TW}=\Lambda e^{\phi _{TW}-V x}$ for any $\Lambda \in {\mathbb {R}}$. The value of $\Lambda =\Lambda (V)$ can be determined by the provision that $\int _{-1/2}^{1/2}m\,{\text {d}}x=1$, and the value of $\phi _{TW}$ therefore satisfies

98 $$\begin{aligned} {\left\{ \begin{array}{ll} -Z\phi _{TW}''+\phi _{TW}=P(V)\Lambda (V)e^{\phi _{TW}(x)-Vx} &  -1/2<x<1/2\\ \phi _{TW}(-1/2)=\phi _{TW}(1/2) &  \\ \phi _{TW}'(-1/2)=\phi _{TW}'(1/2)=V. &  \end{array}\right. } \end{aligned}$$

Note that (98) has three boundary conditions: not only must $\phi _{TW}$ satisfy periodic boundary conditions, but also $\phi _{TW}'(\pm 1/2)=V$. Thus, *P*(*V*) is selected so that $\phi _{TW}$ can satisfy this extra condition.

Solutions to (98) may be approximated asymptotically in small *V* as shown in the following Lemma:

### Lemma 5.2

Let $Z>0$ with $Z\ne 1/12$. Suppose that $m_{TW}$, $\phi _{TW}$ and *P*(*V*) solve (97). In small *V*, *P*(*V*) and $m_{TW}$ have the asymptotic forms

99 $$\begin{aligned} P(V)&=P_0+V^2 P_2+O(V^4)\end{aligned}$$

100 $$\begin{aligned} m_{TW}(x)&=1+Vm_1(x)+V^2m_2(x)+O(V^3), \end{aligned}$$

where

- $P_0$ is a nontrivial solution to (96)
- $P_2$ is given by
  101 $$\begin{aligned} P_2=\frac{P_0 \left( 6 P_0^6-15 P_0^5-3 P_0^4 (56 Z-5)+P_0^3 (514 Z-6)-1044 P_0^2 Z+72 P_0 Z (55 Z-1)+5280 Z^2\right) }{288 (P_0-1)^4 \left( P_0^2-12 Z\right) }.\nonumber \\ \end{aligned}$$
- $m_1$ is given by
  102 $$\begin{aligned} m_1(x)=\frac{x}{P_0-1}-\frac{P_0 \sqrt{\frac{P_0^3-P_0^2+4 Z}{\left( P_0-1\right) P_0^2}} \sin \left( \frac{\sqrt{P_0-1} x}{\sqrt{Z}}\right) }{2 P_0-2} \end{aligned}$$
- $m_2$ is given by
  103 $$\begin{aligned}  &   m_2(x)=A+Bx^2+(C+{\text {d}}x^2)\cos \left( \frac{\sqrt{P_0-1}}{\sqrt{Z}}x\right) \nonumber \\  &   \quad +Ex\sin \left( \frac{\sqrt{P_0-1}}{\sqrt{Z}}x\right) +F\cos \left( \frac{2\sqrt{P_0-1}}{\sqrt{Z}}x\right) \end{aligned}$$
  with
  104 $$\begin{aligned} A&=\frac{-6 P_0 Z+P_0+24 Z-1}{24 \left( P_0-1\right) {}^4}\end{aligned}$$
  105 $$\begin{aligned} B&=\frac{12-12 P_0}{24 \left( P_0-1\right) {}^4}\end{aligned}$$
  106 $$\begin{aligned} C&=\frac{\left( P_0 (28 Z-3)+3 P_0^2-60 Z\right) \sqrt{\frac{P_0^3-P_0^2+4 Z}{Z}}}{96 \left( P_0-1\right) {}^4}\end{aligned}$$
  107 $$\begin{aligned} D&=-\frac{P_0 \sqrt{\frac{P_0^3-P_0^2+4 Z}{Z}}}{8 \left( P_0-1\right) {}^3}\end{aligned}$$
  108 $$\begin{aligned} E&=\frac{\left( 4-3 P_0\right) \sqrt{Z} \sqrt{\frac{P_0^3-P_0^2+4 Z}{Z}}}{8 \left( P_0-1\right) {}^{7/2}}\end{aligned}$$
  109 $$\begin{aligned} F&=\frac{\left( 3-4 P_0\right) \left( P_0^3-P_0^2+4 Z\right) }{48 \left( P_0-1\right) {}^4}. \end{aligned}$$

### Proof

We first introduce expansions for $\Lambda (V)$ and $\phi _{TW}$:

110 $$\begin{aligned} \Lambda (V)&=\Lambda _0+V^2\Lambda _2 +O(V^4),\end{aligned}$$

111 $$\begin{aligned} \phi _{TW}(x)&=\phi _0(x)+V\phi _1(x)+V^2\phi _2(X)+V^3\phi _3(x)+O(V^4). \end{aligned}$$

Observe that neither the expansion for *P* (99) nor the expansion for $\Lambda$ (110) have terms that are of odd order in *V*. This is because we expect symmetry in traveling wave solutions with respect to the sign of *V*. Therefore, *P*(*V*) and $\Lambda (V)$ are even functions of *V* and for $m_{TW}(x)$ and $\phi _{TW}(x)$, the transformation $V\mapsto -V$ is equivalent to $x\mapsto -x$.

Since $m_{TW}(x)=\Lambda (V)e^{\phi _{TW}(x)-V x}$, we may expand the exponential to obtain $1=\Lambda _0e^{\phi _0}$, $m_1(x)=\Lambda _0e^{\phi _0}(\phi _1-x)$ and

112 $$\begin{aligned} m_2(x)=\Lambda _2 e^{\phi _0}+\frac{1}{2} \Lambda _0 e^{\phi _0} \left( x^2-2 x \phi _1+\phi _1^2+2 \phi _2\right) . \end{aligned}$$

We conclude that $\phi _0$ is constant and $\Lambda _0=e^{-\phi _0}$. Substituting the expansion (111) into (98) and comparing terms of like order in *V*, we obtain $\phi _0=P_0$ in zeroth order and the following differential equations in first through third order:

113 $$\begin{aligned} {\left\{ \begin{array}{ll} -Z\phi _1''+(1-P_0)\phi _1=-P_0x &  -1/2<x<1/2\\ \phi _1(1/2)=\phi _1(-1/2) &  \\ \phi _1'(1/2)=\phi _1'(-1/2)=1 \end{array}\right. } \end{aligned}$$

114 $$\begin{aligned} {\left\{ \begin{array}{ll} -Z\phi _2''+(1-P_0)\phi _2=P_2+P_0e^{P_0}\Lambda _2+\frac{P_0}{2}(\phi _1-x)^2 &  -1/2<x<1/2\\ \phi _2(1/2)=\phi _2(-1/2) &  \\ \phi _2'(1/2)=\phi _2'(-1/2)=0 \end{array}\right. } \end{aligned}$$

115 $$\begin{aligned} {\left\{ \begin{array}{ll} -Z\phi _3''+(1-P_0)\phi _3=\left( \phi _1-x\right) \\ \qquad \qquad \left( P_0 \left( \Lambda _2 e^{P_0}+\phi _2\right) +P_2\right) \\ \qquad \qquad +\frac{1}{6} P_0 \left( \phi _1-x\right) {}^3 &  -1/2<x<1/2\\ \phi _2(1/2)=\phi _2(-1/2) &  \\ \phi _2'(1/2)=\phi _2'(-1/2)=0. \end{array}\right. } \end{aligned}$$

If $P_0=1$, then the solution to (113) is

116 $$\begin{aligned} \phi _1=\frac{x^3}{6Z}-\frac{x}{24Z}+\beta \end{aligned}$$

for some $\beta \in {\mathbb {R}}$. To satisfy $\phi _1'(\pm 1/2)=1$, we must have $Z=1/12$. This is a contradiction, so $P_0\ne 1$.

If $P_0\ne 1$, then the solution to (113) is

117 $$\begin{aligned} \phi _1(x)=\frac{P_0x}{P_0-1}-\frac{1}{2}\frac{P_0}{P_0-1}\csc \left( \frac{\sqrt{P_0-1}}{2\sqrt{Z}}\right) \sin \left( \frac{\sqrt{P_0-1}}{2\sqrt{Z}}x\right) . \end{aligned}$$

In order to satisfy the additional condition $\phi _1'(\pm 1/2)=1$, $P_0$ must solve (96). Therefore, (102) is obtained as $m_1(x)=\phi _1(x)-x$.

In (114), $\Lambda _2$ is determined by the condition that

118 $$\begin{aligned} \frac{d^2}{dV^2}\int _{-1/2}^{1/2}m_{TW}(x)\,{\text {d}}x=\frac{d^2}{dV^2}\int _{-1/2}^{1/2}\Lambda (V)e^{\phi _{TW}-Vx}\,{\text {d}}x=0. \end{aligned}$$

The value of $\Lambda _2$ is

119 $$\begin{aligned} \Lambda _2=-e^{-P_0}P_2-\frac{e^{-P_0} \left( 3 P_0^2-60 Z+2\right) }{48 \left( P_0-1\right) {}^2}. \end{aligned}$$

The solution to (114) can be found using elementary methods and has the form

120 $$\begin{aligned}  &   \phi _2(x)=P_2+a_0+a_2x^2+(b_0+b_2x^2)\cos \left( \frac{\sqrt{P_0-1}}{\sqrt{Z}}x\right) \nonumber \\  &   \quad +c_1x\sin \left( \frac{\sqrt{P_0-1}}{\sqrt{Z}}x\right) +d_0\cos \left( \frac{2\sqrt{P_0-1}}{\sqrt{Z}}x\right) \end{aligned}$$

where

121 $$\begin{aligned} a_0&=\frac{P_0^2 (1-30 Z)+P_0 (48 Z-1)}{24 \left( P_0-1\right) {}^4}\end{aligned}$$

122 $$\begin{aligned} a_2&=-\frac{P_0}{2 \left( P_0-1\right) {}^3}\end{aligned}$$

123 $$\begin{aligned} b_0&=\frac{\left( P_0 (28 Z-3)+3 P_0^2-60 Z\right) \sqrt{P_0^3-P_0^2+4 Z}}{96 \left( P_0-1\right) {}^4 \sqrt{Z}}\end{aligned}$$

124 $$\begin{aligned} b_2&=-\frac{P_0 \sqrt{P_0^3-P_0^2+4 Z}}{8 \left( P_0-1\right) {}^3 \sqrt{Z}}\end{aligned}$$

125 $$\begin{aligned} c_1&=\frac{P_0 \sqrt{P_0^3-P_0^2+4 Z}}{8 \left( P_0-1\right) {}^{7/2}}\end{aligned}$$

126 $$\begin{aligned} d_0&=\frac{-4 P_0 Z-P_0^4+P_0^3}{48 \left( P_0-1\right) {}^4}. \end{aligned}$$

Note that the only dependence on $P_2$ in $\phi _2$ is the leading term—none of the other coefficients depend on $P_2$. Therefore, we write $\phi _2=P_2+\tilde{\phi }_2$, where $\tilde{\phi }_2$ is independent of $P_2$. We similarly write $\Lambda _2=-e^{-P_0}P_2+\tilde{\Lambda }_2$.

In third order, we do not need to find an explicit solution $\phi _3$. Instead, we divide the right hand side of the differential equation in (115) to separate terms that explicitly depend on $P_2$ form those that do not:

127 $$\begin{aligned} -Z\phi ''_3+(1-P_0)\phi _3=P_2f(x)+g(x), \end{aligned}$$

where $f(x)=\phi _1-x$ and $g(x)=P_0 \left( \phi _1-x\right) \left( e^{P_0} \tilde{\Lambda }_2+\tilde{\phi }_2\right) +\frac{1}{6} P_0 \left( \phi _1-x\right) {}^3$. The three boundary conditions that $\phi _3$ must satisfy (periodic boundary conditions with $\phi _3'(\pm 1/2)=0$) determine $P_2$, which we show as follows. Let $U(x)=\sin \left( x\sqrt{P_0-1}/\sqrt{Z}\right)$. Then

128 $$\begin{aligned} \int _{-1/2}^{1/2}(P_2f(x)+g(x))U(x)\,{\text {d}}x&=\int _{-1/2}^{1/2}(-Z\phi _3''+(1-P_0)\phi _3)U(x)\,{\text {d}}x \end{aligned}$$

129 $$\begin{aligned}&=-Z\phi _3'U(x)\big |_{-1/2}^{1/2}+Z\phi _3U'\big |_{-1/2}^{1/2}\nonumber \\&\quad +\int _{-1/2}^{1/2}\phi _3(-ZU''+(1-P_0)U)\,{\text {d}}x\end{aligned}$$

130 $$\begin{aligned}&=0. \end{aligned}$$

Therefore, we may explicitly calculate

131 $$\begin{aligned} \begin{aligned} P_2&=-\frac{\int _{-1/2}^{1/2}g(x)U(x)\,{\text {d}}x}{\int _{-1/2}^{1/2}f(x)U(x)\,{\text {d}}x}\\&=\frac{\begin{array}{c}P_0 (6 P_0^6-15 P_0^5-3 P_0^4 (56 Z-5)\\ +P_0^3 (514 Z-6)-1044 P_0^2 Z+72 P_0 Z (55 Z-1)+5280 Z^2)\end{array}}{288 (P_0-1)^4 \left( P_0^2-12 Z\right) }. \end{aligned} \end{aligned}$$

Therefore, substituting (131) into (120), and then into (112), we obtain (103). $\square$

### Remark 5.3

The existence of this bifurcation, demonstrated for the 1D problem in [79, 80], was shown for the 2D problem in [86] (see also [87]). The value of $P_2$ in (101) is identical to the result given in [80] (see Appendix D).

### Remark 5.4

Lemma 5.2 gives the asymptotic form of traveling wave solutions if they exist, but it does not prove that such solutions exist. To prove existence, we have Theorem 5.5 below. In fact, many such traveling wave solutions exist, each corresponding to a different solution to (96). Lemma 5.2 holds for any of these solutions (other than the trivial solution $P_0=1$), but going forward, we reserve $P_0$ to refer to the smallest nontrivial solution to (96).

**Fig. 3:**
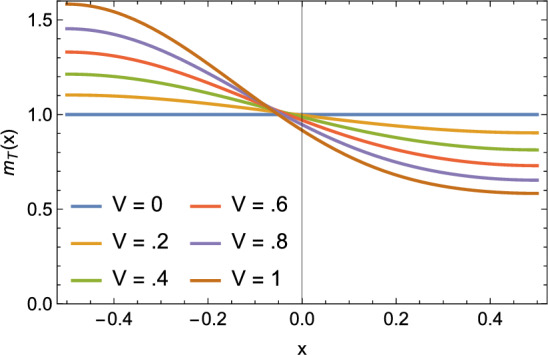
Myosin density of traveling waves for the smallest nontrivial solution $P_0$ to (96). Traveling waves with low velocity have nearly constant myosin density ($m_{TW}\approx 1$), but traveling waves with higher velocity are increasingly asymmetric

A plot of the asymptotic approximation of $m_{TW}(x)$ for the smallest nontrivial solution $P_0$ to (96) given by Lemma 5.2 for several values of *V* is given in Fig. 3.

We can now prove the following result (see also [80]):

### Theorem 5.5

Let $Z>0$ with $Z\ne 1/12$ and suppose $P_0$ is the smallest positive nontrivial solution to (96). Then there exists $V^*>0$ and a continuous function $P_{TW}:(-V^*,V^*)\rightarrow {\mathbb {R}}$ such that for each $V\in (-V^*,V^*)$, there exists a family traveling wave solutions $(m_{TW},\phi _{TW},Vt+c_0)$ of velocity *V* to (34)-(39) with $P=P_{TW}(V)$ and $c_0\in {\mathbb {R}}$. Moreover, $P_{TW}(0)=P_0$ and $m_{TW}$ as a function of *V* is continuously differentiable function from $(-V^*,V^*)$ to $H^2(-1/2,1/2)$.

The parameter $V^*$ in Theorem 5.5 is the (not explicitly known) largest velocity for which traveling waves must exist. That is, the bifurcation of stationary solutions to traveling waves is a strictly local result in a neighborhood of $(m,V)=(1,0)$. The main tool to prove this theorem will the be the CR theorem [25], which we quote in Appendix B.

Essentially, the CR theorem gives conditions under which an equation of the form ${\mathcal {F}}(x,t)=0$ has two families of solutions: a *trivial branch* where $x=0$ and *t* parameterizes the family, and a *nontrivial branch* where *x* and *t* are both parameterized by a new parameter *s*, and the two families meet at $(x,t)=(0,0)$. In Theorem 5.5, the trivial branch corresponds to the stationary homogeneous solution $m=1$ for any value of *P*. The nontrivial branch corresponds to the traveling wave solutions parameterized by their velocity and with activity parameter $P=P_{TW}(V)$. The two families of solutions meet at $P=P_{TW}(0)=P_0$ satisfying (96).

### Proof of Theorem 5.5

Given $Z>0$ with $Z\ne 1/12$, let $P=P_0$ be a nontrivial solution to (96). Let

132 $$\begin{aligned} X=\left\{ \mu \in H^2(-1/2,1/2):\mu '(-1/2)=\mu '(1/2)=0,\int _{-1/2}^{1/2}\mu (x)\,{\text {d}}x=0\right\} \nonumber \\ \end{aligned}$$

and

133 $$\begin{aligned} Y=\left\{ s\in L^2(-1/2,1/2)\times {\mathbb {R}}:\int _{-1/2}^{1/2}s(x)\,{\text {d}}x=0\right\} . \end{aligned}$$

Define $${\mathcal {F}}:X\times {\mathbb {R}}\rightarrow Y$$ by

134 $$\begin{aligned} {\mathcal {F}}(\mu ,\tau )=\mu ''+\phi '(1/2)\mu '-((\mu +1)\phi ')', \end{aligned}$$

where $\phi$ is satisfies $-Z\phi ''+\phi =(P_0+\tau )(1+\mu )$ with periodic boundary conditions in $(-1/2,1/2)$. Observe that

135 $$\begin{aligned} m(x,t)=\mu (x-\phi '(1/2)t,t)+1 \end{aligned}$$

is a traveling wave solution to (17) if and only if $F(\mu ,\tau )=0$. If $\phi '(1/2)=0$, then *m* is a stationary solution, i.e., a traveling wave with velocity 0.

We will show that ${\mathcal {F}}$ satisfies the properties required for the validity of the CR theorem, see Appendix B.

- It is clear that ${\mathcal {F}}(0,\tau )=0$ for all $\tau$.
- It is also clear that ${\mathcal {F}}$ is twice continuously differentiable.
- The linearization of ${\mathcal {F}}$ in $\mu$ at $(\mu ,\tau )=(0,0)$ is
  136 $$\begin{aligned} 0=D_{\mu }{\mathcal {F}}(0,0)u=u''-\psi '' \end{aligned}$$
  where $\psi$ satisfies $-Z\psi ''+\psi =P_0u$ with periodic boundary conditions on $(-1/2,1/2)$. Note that this is the operator $S_C$ in (48). To show that the third hypothesis of the CR theorem is satisfied, we need to show two things:
  (i) There exists a unique (up to multiplicative constant) nonzero solution $u_0\in X$ to $D_\mu {\mathcal {F}}(0,0)u_0=0$, and
  (ii) there exists a co-dimension one subspace $X'$ of *X* such that if $w\in X'$, then there exists a solution $u\in X$ to $D_\mu {\mathcal {F}}(0,0)u=w$.
  First we show (i). Consider the function
  137 $$\begin{aligned} u_0(x)=\frac{\sqrt{Z} \text {sech}\left( \frac{\sqrt{1-P_0}}{2 \sqrt{Z}}\right) \sinh \left( \frac{\sqrt{1-P_0} x}{\sqrt{Z}}\right) }{(1-P_0)^{3/2}}+\frac{x}{P_0-1}. \end{aligned}$$
  Observe that $u_0'(\pm 1/2)=0$ and $\int _{-1/2}^{1/2}u_0(x)\,{\text {d}}x=0$, so $u_0\in X$. Moreover, one may check that provided $P_0$ satisfies (96), then $D_{\mu }{\mathcal {F}}(0,0)u_0=0$. Now suppose that $u_1$ and $u_2$ are both nonzero solutions to (136). Let $\psi _i$ solve $-Z\psi _i''+\psi _i=P_0u_i$ with periodic boundary conditions on $(-1/2,1/2)$ for $i=1,2$. Observe that for each *i*, since the second derivatives of $u_i$ and $\psi _i$ are equal, we have
  138 $$\begin{aligned} u_i-\psi _i=\alpha _i x-\beta _i \end{aligned}$$
  We also observe that
  139 $$\begin{aligned} \int _{-1/2}^{1/2}\psi _i\,{\text {d}}x=P_0\int _{-1/2}^{1/2}u_i\,{\text {d}}x+Z\int _{-1/2}^{1/2}\psi _i''\,{\text {d}}x=0. \end{aligned}$$
  Therefore, $\beta _1=\beta _2=0$. Now, suppose for some *i*, $\psi _i'(\pm 1/2)=0$. Then $\alpha _i=0$ and $u_i=\psi _i$. Thus, $u_i$ satisfies $-Zu_i''+u_i=P_0u_i$ with periodic boundary conditions. We conclude that $u_i$ is an eigenvector of the second derivative operator with eigenvalue $(1-P_0)/Z$. The eigenvalues of the second derivative operator on *X* are $-n^2\pi ^2$ for positive integers *n*. But since $P_0$ and *Z* must satisfy (96) and $P_0\ne 1$, it is clear that $(1-P_0)/Z\ne -n^2\pi ^2$, so we have arrived at a contradiction. Therefore, $\psi _i'(\pm 1/2)\ne 0$, and we may assume without loss of generality that the $u_i$ are scaled such that $\psi _i'(\pm 1/2)=1$ for $i=1,2$, so $\alpha _i=-1$. Then $u_i=\psi _i-x$. Let $w=u_1-u_2$. Then
  140 $$\begin{aligned} w=(\psi _1-x)-(\psi _2-x)=\psi _1-\psi _2. \end{aligned}$$
  Thus, *w* satisfies $-Zw''+w=P_0w$ with periodic boundary conditions on $(-1/2,1/2)$. Once again, since $(1-P_0)/Z\ne -n^2\pi ^2$, the only solution is $w=0$. We conclude that $u_1=u_2$ and $u_0$ is unique up to a multiplicative constant.
  Now we show (ii). Observe that since $\psi$ satisfies $\psi ''=(P_0u-\psi )/Z$, we may write
  141 $$\begin{aligned} D_m{\mathcal {F}}(0,0)u=u''-\frac{P_0}{Z}u+\frac{1}{Z}\psi . \end{aligned}$$
  We may abstract this operator as $D_m{\mathcal {F}}(0,0)=B+K$ where $Bu=u''-(P_0/Z)u$ and $K:u\mapsto \psi /Z$. We make two observations. First, *K* is a bounded operator with respect to the $H^2$ norm and a compact operator with respect to the $L^2$ norm. However, since we will only apply *K* to $u\in X\subset H^2(-1/2,1/2)$, we may restrict to domain of *K* to *X*, and then by the Rellich–Kondrachov Theorem, it is a compact operator in the $H^2$ norm. Second, the operator *B* is invertible on *X* and its inverse $B^{-1}$ is bounded. Therefore, we may write
  142 $$\begin{aligned} I-B^{-1}D_\mu {\mathcal {F}}(0,0)=I-B^{-1}(B+K)=-B^{-1}K \end{aligned}$$
  and
  143 $$\begin{aligned} I-D_\mu {\mathcal {F}}(0,0)B^{-1}=I-(B+K)B^{-1}=-KB^{-1}. \end{aligned}$$
  Since $B^{-1}$ is bounded and *K* is compact, the operators $B^{-1}K$ and $KB^{-1}$ are compact in $H^2$. We conclude that $D_\mu {\mathcal {F}}(0,0)$ is a Fredholm operator on *X*. We recall that the index of a Fredholm operator is the difference between the dimension of its kernel and the codimension of its range. We also recall that the index of a self-adjoint operator is zero. Since *B* and *K* are both self-adjoint over $L^2([-1/2,1/2])$, so is $D_\mu {\mathcal {F}}(0,0)$. Therefore, the codimension of the range of $D_\mu {\mathcal {F}}(0,0)$ is equal to the dimension of the kernel, which we have just proved is 1.
- Finally, we must show that $D_{\mu s}{\mathcal {F}}(0,0)u_0$ is not in the range of the operator $D_{\mu }{\mathcal {F}}(0,0)$. Observe that since $D_\mu {\mathcal {F}}(0,0)$ is self-adjoint, its image is orthogonal to its kernel. That is, for any $u\in X$, $\langle D_\mu {\mathcal {F}}(0,0)u,u_0\rangle _{L^2}=0$. It is therefore sufficient to show that $\langle D_{\mu s} {\mathcal {F}}(0,0)u_0,u_0\rangle _{L^2}\ne 0$. The mixed second derivative is
  144 $$\begin{aligned} D_{\mu s} {\mathcal {F}}(0,0)u=u''-\tilde{\psi }'' \end{aligned}$$
  where $\tilde{\psi }$ satisfies $-Z\tilde{\psi }''+\tilde{\psi }=u$. Therefore, $\tilde{\psi }=\psi /P_0$. We conclude that
  $$\begin{aligned}&\langle D_{\mu s}{\mathcal {F}}(0,0)u_0,u_0\rangle _{L^2}=\int _{-1/2}^{1/2}\left( u_0''-\frac{\psi _0''}{P_0}\right) u_0\,{\text {d}}x\\&\quad =\int _{-1/2}^{1/2}\left( 1-\frac{1}{P_0}\right) u_0u_0''\,{\text {d}}x=-\int _{-1/2}^{1/2}\left( 1-\frac{1}{P_0}\right) (u_0')^2\,{\text {d}}x. \end{aligned}$$
  Since $P_0\ne 1$, $\langle D_{\mu s}{\mathcal {F}}(0,0)u_0,u_0\rangle _{L^2}\ne 0$ (it is also interesting to note that this is why bifurcation does not occur when $P_0=1$).

Since we now checked that all the hypotheses of the CR theorem hold in a neighborhood of $(\mu ,\tau )=(0,0)$, the only solutions to ${\mathcal {F}}(\mu ,\tau )=0$ are $\mu =0$ plus a smooth family of solutions $(\mu (s),\tau (s))$ parameterized by *s* in some small interval $(-s^*,s^*)$ with $\mu (s)\not \equiv 0$. Moreover, these two families of solutions meet at (0, 0). Indeed, let $m_{TW}(s)=1+\mu (s)$ and $P_{TW}(s)=P_0+\tau (s)$. Moreover, $m_{TW}:(-s^*,s^*)\rightarrow H^2(-1/2,1/2)$ is continuously differentiable.

Since all solutions to ${\mathcal {F}}=0$ are traveling waves with some velocity (or stationary solutions if the velocity is zero), it only remains to show that $m_{TW}(s)$ and $P_{TW}(s)$ may be reparameterized (at least locally near $s=0$) by velocity. Let *V*(*s*) be the velocity of $m_{TW}(s)$. It is sufficient to show that $V'(0)\ne 0$. Let $\phi _{TW}(s)$ satisfy $-Z\phi _{TW}''+\phi _{TW}=P_{TW}(s)m_{TW}(s)$ with periodic boundary conditions on $(-1/2,1/2)$. Then $V(s)=\partial _x\phi _{TW}(s)|_{x=1/2}$. Therefore, $V'(0)$ is $\psi '(1/2)$ where $\psi$ solves

145 $$\begin{aligned}  &   -Z\psi ''+\psi =\frac{\partial }{\partial s}P_{TW}(s)m_{TW}(s)\Big |_{s=0}=\frac{\partial P_{TW}}{\partial s}(0)m_{TW}(0)\nonumber \\  &   \qquad +P_{TW}(0)\frac{\partial m_{TW}}{\partial s}(0)=\frac{\partial P_{TW}}{\partial s}(0)+P_0\frac{\partial \mu }{\partial s}(0), \end{aligned}$$

with periodic boundary conditions. Since ${\mathcal {F}}$ is twice differentiable, $\mu (s)$ is also continuously differentiable and $\mu '(0)$ spans the null space of ${\mathcal {F}}_\mu (0,0)$. Without loss of generality, we may assume that $\mu (s)$ is parameterized such that

146 $$\begin{aligned} \frac{d\mu }{ds}(0)=\mu _0. \end{aligned}$$

Let $\psi _0$ solve $-Z\psi _0''+\psi _0=P_0\mu _0$ with periodic boundary conditions on $(-1/2,1/2)$. Then $\psi _0$ and $\psi$ differ by a constant, so $V'(0)=\psi _0'(1/2)$. We may explicitly calculate $\psi _0'(1/2)=1$, so $V'(0)\ne 0$. Therefore, we may smoothly reparameterize $m_{TW}$ and $P_{TW}$ by *V* for *V* in some small interval $(-V^*,V^*)$ such that $m_{TW}:(-V^*,V^*)\rightarrow {\mathbb {H}}^2(-1/2,1/2)$ is continuously differentiable. $\square$

### Remark 5.6

As a result of Theorem 5.5, the map $m_{TW}:(-V^*,V^*)\rightarrow {\mathbb {H}}^2(-1/2,1/2)$ is continuously differentiable. This means that $m_{TW}':(-V^*,V^*)\rightarrow H^1(-1/2,1/2)$ is also continuously differentiable. Since $\phi _{TW}$ is a bounded linear function of $m_{TW}$, it is also a continuously differentiable function from $(-V^*,V^*)$ to $H^2(-1/2,1/2)$. Thus, we can say that each of $m_{TW}$, $m_{TW}'$, $m_{TW}''$, $\phi _{TW}$, $\phi _{TW}'$, and $\phi _{TW}''$ are all continuously differentiable as functions from $(-V^*,V^*)$ to $L^2 (-1/2,1/2)$.

### Remark 5.7

While Theorem 5.5 proves the existence of a bifurcating branch of traveling wave solutions that meets the branch of stationary solutions at $P=P_0$, the smallest positive nontrivial solution to (96), the exact same proof would prove the existence of bifurcating branch of traveling waves emerging from any other positive nontrivial solution to (96). Our interest in the “first” branch of traveling wave solutions arises because this is the only family that is exponentially stable (as we shall see in Sect. 6). Thus, among all families of traveling waves, the notations $m_{TW}$, $\phi _{TW}$, and $P_{TW}$ are used to denote this “first” family.

Given $Z>0$, the condition (96) satisfied by $P_0$ has (potentially) infinitely many solutions. Therefore, Theorem 5.5 proves the existence of not just one, but infinitely many families of traveling wave solutions, each bifurcating from the stationary solution for a different solution *P* to (96). In Sect. 4, we observed that for $P/Z<\pi ^2$, the eigenvalues of the linearization $S_C$ of model *C* about the stationary solution are all negative. In the proof of Theorem 5.5, we observe that if *P* satisfies (96), $S_C$ has a zero eigenvalue. We conclude that as *P*/*Z* increases from $\pi ^2$, each solution of (96) corresponds to one of the eigenvalues of $S_C$ becoming positive. Therefore, we conjecture that for all families of traveling waves except those bifurcating from the smallest solution of (96), the linearization of model C about these traveling waves has some positive eigenvalues, and therefore these traveling waves are unstable. The only traveling wave solutions that may be stable are those bifurcating from the smallest solution to (96). Therefore, when using the notation $P_0$, we refer to this value. The following lemma shows the existence of this smallest solution and provides the illuminating estimate that, for large *Z*, $P_0/Z\ge \pi ^2$ with equality in the limit $Z\rightarrow \infty$.

### Lemma 5.8

Suppose that $P_0=P_0(Z)$ is the smallest nontrivial positive solution to (96) whenever such a solution exists. Then $P_0(Z)$ exists for all $Z>0$ except $Z=1/12$ and in large *Z*, $P_0(Z)$ expands as

147 $$\begin{aligned} P_0(Z)=\pi ^2Z+1-\frac{8}{\pi ^2}+O(1/Z). \end{aligned}$$

### Proof

First suppose $0<Z<1/12$. Let $v=\sqrt{1-P_0}/(2\sqrt{Z})$. Then *v* and *Z* satisfy

148 $$\begin{aligned} k_1(z):=\frac{v-\tanh (v)}{4v^3}=Z. \end{aligned}$$

It is easy to show that $k_1$ is continuous on $(0,\infty )$, $\lim _{v\rightarrow 0^+}k_1(v)=1/12$, $\lim _{v\rightarrow \infty }k_1(v)=0$, and $k_1$ is monotonically decreasing. Thus, for any $Z\in (0,1/12)$, there exists a unique $v\in (0,\infty )$ satisfying (148). Therefore, $P_0(Z)=\tanh (v)/v\in (0,1)$ is uniquely determined.

Now suppose $Z>1/12$. Write (96) as

149 $$\begin{aligned} \tan \left( \frac{\sqrt{P_0-1}}{2\sqrt{Z}}\right) =\frac{P_0\sqrt{P_0-1}}{2\sqrt{Z}} \end{aligned}$$

and let $w=\sqrt{P_0-1}/(2\sqrt{Z})$. Then *w* and *Z* satisfy

150 $$\begin{aligned} k_2(w)=\frac{\tan (w)-w}{4w^3}=Z. \end{aligned}$$

Similarly to $k_1$, it is easy to show that $k_2$ is continuous on $(0,\pi /2)$, $\lim _{w\rightarrow 0^+}k_2(w)=1/12$, $\lim _{w\rightarrow \pi /2^-}k_2(w)=\infty$, and $k_2$ is monotonically increasing on $(0,\pi /2)$. Thus, for any $Z\in (1/12,\infty )$, there exists a unique $w\in (0,\pi /2)$ satisfying (150). Therefore, $P_0(Z)=\tan (w)/w\in (1,\infty )$. Thus, for all positive *Z* other than $Z=1/12$, (96) has a smallest positive solution other than 1. It should be noted that using a similar line of reasoning, we may show that (150) has a unique solution in each interval of the form $(n\pi /2,(n+2)\pi /2)$ for $n>0$ odd. These correspond to the other (larger than $P_0$) solutions to (96) referenced above.

As $Z\rightarrow \infty$ the corresponding solution *w* to $k_2(w)=Z$ approaches $\pi /2$. Therefore, we expand *w* in large *Z* as $w(Z)=\pi /2+w_1/Z+w_2/Z^2+O(1/Z^3)$. We expand (150) in large *Z* and compare terms of like order in *Z* to obtain $w=\pi /2-2/(Z\pi ^3)+O(1/Z^2)$. Finally, using $P_0=1+4Zw^2$, we have

151 $$\begin{aligned} P_0=\pi ^2Z+\left( 1-\frac{8}{\pi ^2}\right) +O(1/Z). \end{aligned}$$

$$\square $$

## Non-Self-Adjoint Spectral Analysis for Nonlinear Stability of Traveling Waves

In this section we study the nonlinear stability of traveling wave solutions to Model B. As shown in Theorem 5.5, traveling wave solutions of velocity *V* sufficiently small exist provided *P* has the prescribed value $P_{TW}(V)$. Such a traveling wave solution has the form $m(x,t)=m_{TW}(x-Vt)$ with $c=c_0+Vt$ where $m_{TW}$ is a stationary solution to model *C*. For ease of analysis we will study the stability of these solutions in the framework of model C which, as described in Sect. 4, implies stability “up to shifts" of traveling wave solutions to model B.

As in Sect. 4, we describe model C by the dynamical system $\partial _t m=F_C(m)$ with $F_C$ given by (46). Let $m_{TW}$ be the family of traveling wave solutions to (46) guaranteed by Theorem 5.5, and let $P_{TW}$ be the corresponding family of activity parameters, both families parameterized by *V* (that is, $m_{TW}$ is the branch of solutions bifurcating from the smallest positive nontrivial solution to (96)). The main result of this section is the following theorem about the exponential stability of $m_{TW}$:

### Theorem 6.1

There exist $V^*, Z^*>0$ such that if $|V|<V^*$ and $Z>Z^*$, then the traveling wave $m_{TW}$ is exponentially stable in the sense that there exists $\varepsilon , r, M>0$ (depending on *V* and *Z*) such that if $m(\cdot ,t)$ is a solution to (46) with $P=P_{TW}(V)$ and $m(\cdot ,0)=m_0\in H^1(-1/2,1/2)$ satisfying

152 $$\begin{aligned} \Vert m_{TW}-m_0\Vert _{H^1}<\varepsilon , \end{aligned}$$

then

153 $$\begin{aligned} \Vert m(\cdot ,t)-m_{TW}\Vert _{L^2}\le \Vert m_{TW}-m_0\Vert _{L^2}e^{-rt}. \end{aligned}$$

While Theorem 6.1 is written to emphasize that traveling waves are stable if their velocity is small ($|V|<V^*$) and $P=P_{TW}(V)$. However, an equivalent way to write Theorem 6.1 would state that traveling waves are stable if the activity rate *P* is close to $P_0$ (more specifically, $P\in P_{TW}(-V^*,V^*)$) and velocity *V* satisfies $P=P_{TW}(V)$.

To prove Theorem 6.1, we follow the same strategy as proving Theorem 4.1. However, a significant challenge is introduced in that the linearization of $F_C$ about $m_{TW}$ is *not self-adjoint*, meaning that the classical techniques of Sect. 4 (based on self-adjointness of the linearization) no longer apply. Therefore, we develop a new method of proving linear stability based on the Grearhart-Prüss-Greiner (GPG) Theorem.

We decompose $F_C$ as a sum of its linearization $T_C$ about $m_{TW}$ and its “nonlinear part” $\Psi$:

154 $$\begin{aligned} F_C(m_{TW}+u)=T_Cu+\Psi (u). \end{aligned}$$

The linearization about $m_{TW}$ about traveling waves is:

155 $$\begin{aligned} T_Cu:=DF_C(m_{TW})u=u''+\phi '(1/2)m_{TW}'+V u'-(m_{TW}\phi ')'-(u\phi _{TW}')', \nonumber \\ \end{aligned}$$

where $\phi$ and $\phi _{TW}$ satisfy respectively

156 $$\begin{aligned}  &   {\left\{ \begin{array}{ll} -Z\phi ''+\phi =Pu &  -1/2<x<1/2\\ \phi (1/2)=\phi (-1/2)\\ \phi '(-1/2)=\phi '(1/2) \end{array}\right. }\quad \text {and}\nonumber \\  &   {\left\{ \begin{array}{ll} -Z\phi _{TW}''+\phi _{TW}=Pm_{TW} &  -1/2<x<1/2\\ \phi _{TW}(1/2)=\phi _{TW}(-1/2)\\ \phi _{T,x}(-1/2)=\phi _{T,x}(1/2), \end{array}\right. } \end{aligned}$$

and $u\in \tilde{X}^2_C:=\left\{ m\in H^2(-1/2,1/2):m'(\pm 1/2)=0,\,\int _{-1/2}^{1/2}u\,{\text {d}}x=0\right\}$. The coefficient *V* appears because the velocity of the traveling wave is $V=\phi _{TW}'(1/2)$.

Since the nonlinearity in $F_C$ is quadratic (that is, the Keller-Segel term $(m\phi ')'$), the nonlinear part about the traveling wave $m=m_{TW}$ is the same as the nonlinear part about the stationary state $m=1$:

157 $$\begin{aligned} \Psi (u)=F_C(m_{TW}+u)-T_C u=\phi '(1/2)u'-(u\phi ')', \end{aligned}$$

with $\phi$ given in (156). Using the nonlinear part $\Psi$, and letting $u=m-m_{TW}$, we may rewrite the evolution equation (46) as

158 $$\begin{aligned} \partial _t u=T_Cu+\Psi (u). \end{aligned}$$

Similar to Sect. 4, our analysis in this section is focused on proving two key results:

1. 0 is an exponentially stable equilibrium of the linearized problem $u_t=T_Cu$, and
2. Near $m_{TW}$, the linear part of $F_C$ dominates the nonlinear part.

These results are given by Theorem 6.13 and Lemma 6.14, respectively. These two results are the traveling wave analogues of Theorem 4.3 and Lemma 4.9, and therefore once they are proved, the proof of Theorem 6.1 is identical to the proof of Theorem 4.1.

As described above however, a new challenge arises in this linearization: the operator $T_C$ is non-self-adjoint, meaning that the spectral theorem used in the proof of linear stability in Theorem 4.3 no longer applies. Indeed, while, as we have already mentioned in the Introduction, a self-adjoint operator with compact inverse has a basis of eigenvectors, no such basis is guaranteed if the operator is non-self-adjoint operator, meaning that there may be a portion of the domain of the operator hidden from the eigenvectors. Since the action of the operator on this “dark” space cannot be determined from the eigenvectors, it is not sufficient merely to show that all the eigenvalues of the operator have negative real part. Instead, we rely on the GPG theorem [41], which we quote for convenience in Appendix C.

The GPG theorem overcomes the problem with invisibility of a part of the domain by considering not just eigenvalues, but the entire spectrum of the operator. The spectrum of a linear operator *L* is the set of all $\lambda \in {\mathbb {C}}$ so that the operator $\lambda I-L$ does not have a bounded inverse. Note that if $\lambda I-L$ is not invertible because it is not injective (one-to-one), then $\lambda$ is an eigenvalue of *L*.

We recall that if *L* is a finite dimensional linear operator (a matrix), then the rank-nullity theorem applies and $\lambda I-L$ is invertible if and only if it is injective. In the infinite dimensional case, however, a linear operator may be injective but not surjective, and thus not invertible. Even if $\lambda I-L$ is invertible, its inverse may not bounded. Thus, the spectrum of *L* may consist of more than just eigenvalues.

We also recall that if $\lambda I-L$ does have a bounded inverse, $(\lambda I-L)^{-1}$ is called a resolvent operator of *L*, and the set of $\lambda$ such that the resolvent exists (that is the complement of the spectrum) is called the resolvent set. The solution *x*(*t*) to the linear system $x_t=Lx$ can be written in terms of the resolvent via a line integral in the complex plane as an inverse Fourier Transform as

159 $$\begin{aligned} x(t)=\lim _{s\rightarrow \infty }\frac{1}{2\pi i}\int _{w-is}^{w+is}e^{\lambda t}(\lambda I-L)^{-1}x(0)\,d\lambda \end{aligned}$$

for $w\in {\mathbb {R}}$ sufficiently large [41]. Furthermore, if *x*(0) can be written $x(0)=\sum _{n=1}^\infty c_n x_n$ for eigenvectors $x_n$ of *L* with eigenvalues $\lambda _n$, then if $w>\sup _n\text {Re}\,\lambda _n$,

160 $$\begin{aligned} x(t)&=\lim _{s\rightarrow \infty }\frac{1}{2\pi i}\int _{w-is}^{w+is}e^{\lambda t}(\lambda I-L)^{-1}\sum _{n=1}^\infty c_nx_n\,d\lambda \end{aligned}$$

161 $$\begin{aligned}&=\sum _{n=1}^\infty c_nx_n\lim _{s\rightarrow \infty }\frac{1}{2\pi i}\int _{w-is}^{w+is}\frac{e^{\lambda t}}{\lambda -\lambda _n}\,d\lambda \end{aligned}$$

162 $$\begin{aligned}&=\sum _{n=1}^\infty c_nx_n\frac{e^{wt}}{2\pi }\int _{-\infty }^\infty \frac{e^{ist}}{w+is-\lambda _n}\,ds\end{aligned}$$

163 $$\begin{aligned}&=\sum _{n=1}^\infty c_nx_n\frac{e^{wt}}{2\pi }\left( 2\pi e^{(\lambda _n-w)t}\right) \end{aligned}$$

164 $$\begin{aligned}&=\sum _{n=1}^\infty c_ne^{\lambda _nt}x_n. \end{aligned}$$

The crucial observation is that (159) holds even if *x*(0) cannot be written as a sum of eigenvectors (i.e., if the eigenvectors of *L* do not span the domain of *L*) provided the resolvent $((w+is)I-L)^{-1}$ exists for all $s\in {\mathbb {R}}$ and *w* is sufficiently large.

The GRG theorem (formulated in Appendix C) provides conditions on the resolvent and spectrum of *L* such that, via (159), all solutions *x*(*t*) converge to 0 exponentially fast. Then, since the solutions to our linearized problem $u_t=T_Cu$ are $u(t)=S(t)u(0)$, we can conclude $\lim _{t\rightarrow \infty }\Vert u(t)\Vert \le \lim _{t\rightarrow \infty }e^{-\sigma t}\Vert u(0)\Vert =0$ whenever the conditions of the GRG theorem hold. Then 0 is exponentially stable in the linearized system.

In view of the above, to establish linear stability, it remains to be shown that each of the three conditions of the GPG theorem hold for the operator $T_C$. Those are identified in our Appendix C as conditions (i),(ii),(iii) and are also spelled out explicitly below. We will begin with proving condition (ii), then condition (i), and finally condition (iii). Before proceeding with these steps, we first show that $T_C$ is non-self-adjoint.

### Theorem 6.2

There exists $Z^*$, $V^*>0$ such that if $Z>Z^*$ and $0<|V|<V^*$, the operator $T_C$ is non-self-adjoint.

### Proof

As in the proof of Lemma 4.5, we will use the adjoint commutator. We will show that there exists $V^*>0$ and $u_1,u_2\in \tilde{X}^2_C$ so that if $0<|V|<V^*$, then the adjoint commutator *H* for the operator $T_C=T_C(V)$ evaluated at $u_1,u_2$ is nonzero. This shows that $T_C(V)$ is non-self-adjoint.

Let $u_1(x)=\sin (\pi x)$ and $u_2(x)=\cos (2\pi x)$. Both $u_1$ and $u_2$ are in $\tilde{X}^2_C$. For $i=1,2$ et $\psi _i$ satisfy $-Z\psi _i''+\psi _i=P_{TW}(V)u_i$ with periodic boundary conditions in $(-1/2,1/2)$. Then

165 $$\begin{aligned} \begin{aligned} H(u_1,u_2)&=\int _{-1/2}^{1/2}u_1u_2''-u_2u_1''\,{\text {d}}x+\int _{-1/2}^{1/2}m_{TW}'(u_1\psi _2'(1/2)\\&-u_2\psi _1'(1/2))\,{\text {d}}x+V\int _{-1/2}^{1/2}u_1u_2'-u_2u_1'\,{\text {d}}x\\&-\int _{-1/2}^{1/2}u_1(m_{TW}\psi _2')'-u_2(m_{TW}\psi _1')'\,{\text {d}}x\\&-\int _{-1/2}^{1/2}u_1(u_2\phi _{TW}')'-u_2(u_1\phi _{TW}')'\,{\text {d}}x. \end{aligned} \end{aligned}$$

The function $\psi _1,\psi _2$ depend on *V* through $P_{TW}(V)$, but from Lemma 5.2, $P_{TW}'(0)=0$, so $\partial _V\psi _i|_{V=0}=0$. Also from Proposition 5.2, the traveling wave solution satisfy $\partial _V m_{TW}|_{V=0}=m_1$ given by (102) and $\partial _V\phi _{TW}|_{V=0}=m_1(x)+x$. Therefore,

166 $$\begin{aligned} \begin{aligned} \partial _VH(u_1,u_2)|_{V=0}&=\int _{-1/2}^{1/2}u_1u_2''-u_2u_1''\,{\text {d}}x+\int _{-1/2}^{1/2}m_1'(u_1\psi _2'(1/2)\\&-u_2\psi _1'(1/2))\,{\text {d}}x+\int _{-1/2}^{1/2}u_1u_2'-u_2u_1'\,{\text {d}}x\\&-\int _{-1/2}^{1/2}u_1(m_1\psi _2')'-u_2(m_1\psi _1')'\,{\text {d}}x\\&-\int _{-1/2}^{1/2}u_1(u_2\phi _1')'-u_2(u_1\phi _1')'\,{\text {d}}x. \end{aligned} \end{aligned}$$

Since each of the functions $m_1$, $\phi _1$, $u_1$, $u_2$, $\psi _1$, and $\psi _2$ are explicitly known, and using $P_0=\pi ^2Z+O(1)$ from Lemma 5.8, we may explicitly calculate the integrals in (166) and find the asymptotic expansion of the result in large *Z*:

167 $$\begin{aligned} \partial _VH(u_1,u_2)|_{V=0}=-3+O(1/Z). \end{aligned}$$

Therefore, for sufficiently large $Z^*$, if $Z>Z^*$, then $\partial _VH(u_1,u_2)|_{V=0}\ne 0$. Thus, there exists $V^*$ so that if $0<|V|<V^*$, $H(u_1,u_2)\ne 0$. We conclude that for $Z>Z^*$ and $0<|V|<V^*$, $A_{TW}(V)$ is non-self-adjoint. $\square$

**Condition (ii): the resolvent set of **$T_C$ **contains the right half-plane, see appendix C. ** To establish that condition (ii) holds, we prove a sequence of four results. First, we show that the resolvent of $T_C$, if it exists, is compact. Then we show that for some $\lambda _0>0$, there exists a unique weak solution to $(\lambda _0-T_C)u=w$ for each *w*, which implies that the resolvent $(\lambda _0I-T_C)^{-1}$ exists. Next, we use the first two results to show that the spectrum of $T_C$ consists only of its eigenvalues. Finally, we show that all the eigenvalues of $T_C$ have negative real part. Thus, the resolvent set contains all complex numbers with positive real part, and condition (ii) is satisfied.

### Proposition 6.3

Suppose $\lambda \in {\mathbb {C}}$ such that $\lambda I-T_C$ is invertible. Then $(\lambda I-T_C)^{-1}:L^2(-1/2,1/2)\rightarrow L^2(-1/2,1/2)$ is a compact operator.

### Proof

To be compact, $(\lambda I-T_C)^{-1}$ must be bounded. Suppose, to the contrary, that it is unbounded. Then there exist sequences $(v_k)\subset \tilde{X}^2_C$ and $(w_k)\subset L^2(-1/2,1/2)$ such that

168 $$\begin{aligned} (\lambda I-T_C)v_k=w_k,\quad \Vert v_k\Vert _{L^2}=1,\quad \Vert w_k\Vert _{L^2}\le 1/k. \end{aligned}$$

Let $\phi _k$ satisfy $-Z\phi _k''+\phi _k=Pv_k$ with periodic boundary conditions. Then the following sequence is bounded:

169 $$\begin{aligned} \begin{aligned} \langle w_k,v_k\rangle _{L^2}&=\langle (\lambda I-T_C)v_k,v_k\rangle _{L^2}\\&=\lambda \Vert v_k\Vert _{L^2}^2+\Vert v_k'\Vert _{L^2}^2\\&\quad +\langle (V-\phi _{TW}') v_k',v_k\rangle _{L^2}+\langle (\phi _k'(1/2)-\phi _k')m_{TW}',v_k\rangle _{L^2}\\&\quad -\langle \phi _{TW}''v_k,v_k\rangle _{L^2}-\langle \phi _k''m_{TW},v_k\rangle _{L^2}. \end{aligned} \end{aligned}$$

Every term in this sequence is individually bounded due to Proposition 7.1 in Appendix A except possibly $\Vert v_k'\Vert _{L^2}^2$ and $\langle (V-\phi _{TW}') v_k',v_k\rangle _{L^2}$. However, the sum of these terms must be bounded. While the former is quadratic in $\Vert v_k'\Vert _{L_2}$, the latter is at most linear. Therefore, they must both be independently bounded as well.

Since $\Vert v_k\Vert _{L^2}$ and $\Vert v_k'\Vert _{L^2}$ are both bounded, we conclude that $(v_k)$ is bounded with respect to the $H^1$ norm. The remaining arguments giving rise to a contradiction and proving that $(\lambda I-T_C)^{-1}$ is bounded and, moreover, compact, are identical to those in the proof of Lemma 4.7. $\square$

### Proposition 6.4

There exists $V^*>0$ and $\lambda _0\ge 0$ such that for each $w\in X^0$, there exists a unique weak solution $u\in X^1$ to $(\lambda _0I-T_C)u=w$.

### Proof

Define the bilinear form $B:X^1\times X^1\rightarrow {\mathbb {R}}$ by

170 $$\begin{aligned}  &   B[u,v]=\langle u',v'\rangle _{L^2}-\langle (\phi '(1/2)-\phi ')m_{TW}'\nonumber \\  &   \quad +(V-\phi _{TW}')u'-m_{TW}\phi ''-u\phi _{TW}'',v\rangle _{L^2}. \end{aligned}$$

Then $u\in X^1$ is a weak solution to $(\lambda _0I-T_C)u=w$ if and only if $\lambda _0\langle u,v\rangle +B[u,v]=\langle w,v\rangle _{L^2}$ for all $v\in X^1$. We claim that there exist $a,b,V^*>0$ and $\lambda _0\ge 0$ such that if $|V|<V^*$, then

- $$|B[u,v]|\le a\Vert u\Vert _{H^1}\Vert v\Vert _{H^1}$$
- $$b\Vert v\Vert ^2_{H^1}\le B[v,v]+\lambda _0\Vert v\Vert _{L^2}^2$$.

The proof of these facts follows from the Poincaré inequality and the fact that $\Vert \phi _{TW}'\Vert _{L^2}=O(V)$. Therefore, by the Lax-Milgram Theorem, there exists a unique weak solution to $(\lambda _0 I-T_C)u=w$. $\square$

### Proposition 6.5

The spectrum of $T_C$ consists only of its eigenvalues.

### Proof

This proof is essentially showing that the Fredholm alternative applies to $T_C$. Let $\lambda \in {\mathbb {C}}$, and let $\lambda _0$ be defined as in Proposition 6.4. Define $\hat{T}_C=\lambda _0 I-T_C$ and let $\lambda '=\lambda _0-\lambda$. Then $\lambda I-T_C=\hat{T}_C-\lambda 'I$. By Proposition 6.4, $\hat{T}_C$ is invertible, and by Proposition 6.3, $\hat{T}_C^{-1}$ is compact. Therefore, we may apply the Fredholm alternative for compact operators [36] to see that exactly one of the following holds:

- $(I-\lambda '\hat{T}_C^{-1})v=\hat{T}_C^{-1}w$ has a unique solution for each $w\in X^0$,
- $(I-\lambda '\hat{T}_C^{-1})v=0$ has a nontrivial solution.

In either case, we may multiply by $\hat{T}_C$ to see that either $(\lambda I-T_C)v=w$ has a unique solution for all $w\in X^0$ or $(\lambda I-T_C)v=0$. Therefore, either $\lambda I-T_C$ is invertible (with bounded inverse per Proposition 6.3) and therefore $\lambda$ is not in the spectrum, or $\lambda$ is an eigenvalue of $T_C$. Therefore, the spectrum of $T_C$ consists only of its eigenvalues. $\square$

The following lemma shows that all eigenvalues of $T_C$ have negative real part except possibly one. The following Theorem concerns this remaining eigenvalue showing that it too has negative real part, thus proving the desired result.

### Lemma 6.6

For *V* sufficiently small the eigenvalues of $T_C=T_C(V)$ all have negative real part bounded away from 0 except possibly one. Moreover, when $V=0$, all the eigenvalues of $T_V(0)$ are negative (and real) except for a zero eigenvalue with multiplicity 1.

### Proof

The domain of $T_C(V)$ is $\tilde{X}^2_C$, which has the (Schauder) basis ${\mathcal {B}}=\{v_1,v_2,v_3,\cdots \}$ where $v_n(x)=\sin (n\pi x)$ for *n* odd, and $v_n(x)=\cos (n\pi x)$ for *n* even. For each $n,m\in {\mathbb {N}}$, define

171 $$\begin{aligned} a_{mn}=\langle v_m, T_C(V)v_n\rangle _{L^2} \end{aligned}$$

Treating $A=(a_{mn})$ as an “infinite matrix” operator on $\ell ^2$, we see that $\lambda$ is an eigenvalue of $T_C(V)$ if and only if $\lambda /2$ it is an eigenvalue of *A*. In particular, the eigenvalues of *A* and $T_C(V)$ have the same sign.

Many of the terms in $T_C(V)$ vanish as $V\rightarrow 0$. In particular, the traveling waves $m_{TW}$ and $\phi _{TW}$ and their derivatives depend smoothly on *V* in $L^2$ (see Remark 5.6). Moreover, when $V=0$, $m_{TW}$ and $\phi _{TW}$ are both constant in *x* (they are stationary states). Therefore, writing $m_{TW}=1+\tilde{m}_{TW}$, there exists $C_1,V^*>0$ so that if $|V|<V^*$, then

172 $$\begin{aligned} \Vert m_{TW}'\Vert _{L^1},\Vert \phi _{TW}'\Vert _{L^1},\Vert \phi _{TW}''\Vert _{L^1},\Vert \tilde{m}_{TW}\Vert _{L^1}\le C_1|V|. \end{aligned}$$

For each *m*, let $\phi _m$ solve $-Z\phi _m''+\phi _m=P_{TW}(V)v_m$ with periodic boundary conditions in $(-1/2,1/2)$. For each *n*, *m*, define

173 $$\begin{aligned}  &   d_{mn}=\langle v_m,\phi _n'(1/2)m_{TW}'+Vv_n'-\tilde{m}_{TW}\phi _n''\nonumber \\  &   \quad -m_{TW}'\phi _n'-v_n'\phi _{TW}'-v_n\phi _{TW}''\rangle _{L^2}. \end{aligned}$$

From (172) and Lemma 6.8, there exists $C>0$ independent of *m* such that

174 $$\begin{aligned} \sum _{n=1}^\infty |d_{mn}|\le C|V|\quad \text {and}\quad \sum _{m=1}^\infty |d_{mn}|\le C|V|. \end{aligned}$$

Then we may write for each *n*, *m*:

175 $$\begin{aligned} a_{mn}=\langle v_m,T_C(0)v_n\rangle _{L^2}+d_{mn}. \end{aligned}$$

The operator $T_C(0)$ (which is equal to $S_C(P_0)$) is defined by $T_C(0)u=u''-\phi ''$ where $\phi$ solves $-Z\phi ''+\phi =P_0u$ with periodic boundary conditions in $(-1/2,1/2)$. Thus, letting $c_{mn}=\langle v_n,T_C(0)v_m\rangle _{L^2}$, we have $a_{mn}=c_{mn}+d_{mn}$. We can explicitly calculate $c_{mn}$:

176 $$\begin{aligned} c_{mn}={\left\{ \begin{array}{ll} -n^2\pi ^2+\frac{P_0}{Z}\frac{1}{1+\frac{1}{\pi ^2 n^2 Z}} &  n=m\text { even}\\ -n^2\pi ^2+\frac{P_0}{Z}\frac{1}{1+\frac{1}{\pi ^2 n^2 Z}}-\frac{4P_0\coth \left( \frac{1}{2\sqrt{Z}}\right) }{\sqrt{Z}(1+n^2\pi ^2Z)^2} &  n=m \text { odd}\\ 0 &  n\ne m \text { either }m\text { or }n\text { even}\\ -\frac{4 P_0 (-1)^{\frac{m+1}{2}+\frac{n+1}{2}} \coth \left( \frac{1}{2 \sqrt{Z}}\right) }{\sqrt{Z} \left( \pi ^2 m^2 Z+1\right) \left( \pi ^2 n^2 Z+1\right) } &  n\ne m \text { both odd}. \end{array}\right. } \end{aligned}$$

To show that all the eigenvalues of *A* are negative except possibly one of them we will use Theorem 3 of [94], which gives a Gershgorin-type result showing that all eigenvalues of an infinite matrix have negative real part. While possibly not all eignevalues of *A* have negative real part, we will see using Theorem 3 of [94] that all eigenvalues of $D=B-I$ do have negative real part, and that all but one of these eigenvalues has real part less than $-1$, thus proving the desired result.

The specific result of Theorem 3 of [94] is that there are countably many eigenvalues $\hat{\lambda }_n$ of $B=(b_{mn})$ and for each *n*,

177 $$\begin{aligned} |\hat{\lambda }_n-b_{nn}|<Q_n:=\sum _{\begin{array}{c} m=1\\ m\ne n \end{array}}^\infty |b_{mn}|, \end{aligned}$$

provided the following conditions are met:

1. $b_{nn}\ne 0$ for any *n* and $\lim _{n\rightarrow \infty }|b_{nn}|=\infty$.
2. There exists $0<\rho <1$ so that for each odd *n*,
  178 $$\begin{aligned} \frac{Q_n}{|b_{nn}|}<\rho . \end{aligned}$$
3. For each odd *n*, *m* with $n\ne m$, $|b_{nn}-b_{mm}|\ge Q_n+Q_m$
4. For each *m*, $$\sup \{|b_{mn}|:n\in {\mathbb {N}}\}<\infty $$.

We will show that *B* satisfies each of these conditions for small enough $V<V^*$ and large enough *Z*.

1. Observe that
  179 $$\begin{aligned} b_{nn}<-n^2\pi ^2+\frac{P_0}{Z}\frac{1}{\frac{1}{n^2\pi ^2Z}+1}+C|V|-1. \end{aligned}$$
  Let $0<\varepsilon <1/(2+2\pi ^2)$. Using Lemma 5.8, there exists $Z^*$ large enough that for all $Z>Z^*$, $P_0/Z<\pi ^2+\varepsilon /2$. There also exists $V^*>0$ so that if $|V|<V^*$, $C|V|<\varepsilon /2$. Therefore, for large enough *Z*, $b_{nn}<-\pi ^2(n^2-1)-1+\varepsilon <0$. It is clear that $\lim _{n\rightarrow \infty }|b_{nn}|=\infty$.
2. We have
  180 $$\begin{aligned} Q_n=\sum _{\begin{array}{c} m=1\\ m\ne n \end{array}}^\infty |b_{mn}|\le \sum _{\begin{array}{c} m=1\\ m\ne n \end{array}}^\infty |c_{mn}|+\sum _{\begin{array}{c} m=1\\ m\ne n \end{array}}^\infty |d_{mn}| \end{aligned}$$
  If *n* is even, $Q_n\le \sum _{n=1}^\infty |d_{mn}|<C|V|<\varepsilon /2$. If *n* is odd, we can explicitly calculate a convenient upper bound for $Q_n$:
  181 $$\begin{aligned} Q_n&<Q_n+\frac{4 P_0 \coth \left( \frac{1}{2 \sqrt{Z}}\right) }{\sqrt{Z} \left( \pi ^2 n^2 Z+1\right) ^2}\end{aligned}$$
  182 $$\begin{aligned}&\le \sum _{\begin{array}{c} m=1\\ m\text { odd} \end{array}}^\infty \frac{4 P_0 \coth \left( \frac{1}{2 \sqrt{Z}}\right) }{\sqrt{Z} \left( \pi ^2 m^2 Z+1\right) \left( \pi ^2 n^2 Z+1\right) }+\sum _{m=1}^\infty |d_{mn}|\end{aligned}$$
  183 $$\begin{aligned}&\le \frac{P_0}{Z}\frac{1}{1+n^2\pi ^2Z}+C|V|\end{aligned}$$
  184 $$\begin{aligned}&<\frac{\pi ^2+\varepsilon /2}{1+\pi ^2}+\frac{\varepsilon }{2}\quad \text {assuming }Z^*\ge 1. \end{aligned}$$
  We conclude that whether *n* is even or odd, (184) is an upper bound for $Q_n$. We have already seen that for $Z>Z^*$, $b_{nn}<-1+\varepsilon$. Thus, for any *n*,
  185 $$\begin{aligned}  &   \frac{Q_n}{|b_{nn}|}<\frac{\pi ^2+\varepsilon /2}{(1+\pi ^2)(1-\varepsilon )}+\frac{\varepsilon /2}{1-\varepsilon }<\frac{\pi ^2+\frac{1}{4+4\pi ^2}}{(1+\pi ^2)\left( 1-\frac{1}{2+2\pi ^2}\right) }\nonumber \\  &   \qquad +\frac{1}{(4+4\pi ^2)\left( 1-\frac{1}{2+2\pi ^2}\right) }=\frac{2+5 \pi ^2+4 \pi ^4}{2+6 \pi ^2+4 \pi ^4}<1. \end{aligned}$$
  Therefore, letting $\rho =\frac{2+5 \pi ^2+4 \pi ^4}{2+6 \pi ^2+4 \pi ^4}$, the second condition is satisfied.
3. Let $n,m\in {\mathbb {N}}$ be odd with $n\ne m$. One can verify that for any $Z>0$,
  186 $$\begin{aligned} 0<\frac{4\sqrt{Z}\coth \left( \frac{1}{2\sqrt{Z}}\right) }{(1+\pi ^2Z)^2}<1. \end{aligned}$$
  Then if $Z>Z^*$ and $Z>Z^*$,
  187 $$\begin{aligned} |b_{nn}-b_{mm}|&\ge \pi ^2|n^2-m^2|-\frac{P_0}{Z}\left| \frac{1}{\frac{1}{n^2\pi ^2Z}+1}-\frac{1}{\frac{1}{m^2\pi ^2Z}+1}\right| \end{aligned}$$
  188 $$\begin{aligned}&\quad -\left| \frac{4P_0\coth \left( \frac{1}{2\sqrt{Z}}\right) }{\sqrt{Z}(1+n^2\pi ^2Z)^2}-\frac{4P_0\coth \left( \frac{1}{2\sqrt{Z}}\right) }{\sqrt{Z}(1+m^2\pi ^2Z)^2}\right| -2C|V| \nonumber \\ \end{aligned}$$
  189 $$\begin{aligned}&\ge \pi ^2|n^2-m^2|-2\frac{P_0}{Z}-\varepsilon \end{aligned}$$
  190 $$\begin{aligned}&>3\pi ^2-2(\pi ^2-\varepsilon /2)-\varepsilon \end{aligned}$$
  191 $$\begin{aligned}&>\pi ^2-2\varepsilon . \end{aligned}$$
  On the other hand, we have seen that for each *n*, if $Z>Z^*$ and $|V|<V^*$, then $Q_n<1$, so $Q_n+Q_m<2<\pi ^2-2\varepsilon$. Thus condition 3 is satisfied.
4. This is clear.

Thus, the eigenvalues of *B* are enumerated $\hat{\lambda }_1,\hat{\lambda }_2,\hat{\lambda }_3,\cdots$, and for each *n*, $|b_{nn}-\hat{\lambda }_n|<Q_n$. Thus, for $n\ge 2$,

192 $$\begin{aligned} \text {Re}\,\hat{\lambda }_n<b_{nn}+Q_n<-4\pi ^2+\frac{P_0}{V_0}+C|V|<-3\pi ^2+\varepsilon <-1. \end{aligned}$$

Since the eigenvalues of *A* are $\lambda _n=\hat{\lambda }_n+1$, we conclude that all $\lambda _n$ have negative real part bounded away from 0 except for possibly $\lambda _1$. The eigenvalues of $T_C(V)$ are $2\lambda _n$ for $n=1,2,3,\cdots$, so the desired result holds.

In the case $V=0$, the operator $T_C(0)$ is exactly the operator shown to have exactly one zero eigenvalue in the proof of Theorem 5.5. Therefore, $T_C(0)$ has all negative eigenvalues (real because the operator is self-adjoint) except for one zero eigenvalue. $\square$

### Theorem 6.7

There exists $V^*,Z^*>0$ such that if $0<|V|<V^*$ and $Z>Z^*$, then resolvent set of $T_C$ contains $\{z\in {\mathbb {C}}:\text {Re}\,z\ge 0\}$.

### Proof

Due to Proposition 6.5, we need only show that all eigenvalues of $T_C$ have negative real part. Lemma 6.6 gives $V^*$ and $Z^*$ so that if $|V|<V^*$ and $Z>Z^*$, then all but possibly one of the eigenvalues of $T_C(V)$ has negative real part. We also know that when $V=0$, this one eigenvalue is zero. Therefore, we only need to show that for $0<|V|<V^*$, this eigenvalue has negative real part.

Since $T_C$ depends on *V*, both explicitly, and through $m_{TW}$ and $\phi _{TW}$, we write $T_C=T_C(V)$. For the operator $T_C(V)$, the parameter $P=P_{TW}(V)$ is given by Theorem 5.5. We also consider the linearization $S_C(P)$ of *F* about $m=1$ with arbitrary $P>0$. We will make use of Corollary 1.13 and Theorem 1.16 in [26] which from which we conclude the following:

- There exists neighborhoods $U_1,U_2\subset {\mathbb {R}}$ of 0 and $P_0\in {\mathbb {R}}$ respectively and smooth functions $\lambda :U_1\rightarrow {\mathbb {R}}$ and $\mu :U_2\rightarrow {\mathbb {R}}$ such that $\lambda (V)$ is an eigenvalue of $T_C(V)$ and $\mu (P)$ is an eigenvalue of $T_C(P)$, and $\lambda (0)=\mu (P_0)=0$.
- $$\lambda $$ and $$\mu $$ satisfy:
  193 $$\begin{aligned} -\mu '(P_0)\lim _{V\rightarrow 0}\frac{VP_{TW}'(V)}{\lambda (V)}=1. \end{aligned}$$

By Lemma 6.6, $\lambda (0)=0$ is the largest eigenvalue of $T_C(0)$. Since $T_C$ depends smoothly on *V*, so does $\lambda (V)$. Therefore, for small *V*, $\lambda (V)$ is the eigenvalue of $T_C(V)$ with the largest real part. Moreover, for small *V*, $\lambda (V)$ has the same sign as $-VP'(V)\mu '(P_0)$. From Proposition 5.8, $P_{TW}'(0)=0$. For similar reasons, $\lambda '(0)=0$. So after two applications of L’Hôpital’s rule on (193), we obtain $\lambda (V)=\frac{1}{2}\lambda ''(0)V^2+O(V^3)$ and

194 $$\begin{aligned} \lambda ''(0)=-2P_{TW}''(0)\mu '(P_0). \end{aligned}$$

Therefore, if $P_{TW}''(0)\mu '(P_0)>0$, then there exists $V^*>0$ such that if $0<|V|<V^*$, then $\lambda (V)<0$. We will show that for sufficiently large *Z*, both $P_{TW}''(0)$ and $\mu '(P_0)$ are positive, thus proving the desired result.

First we show that $\mu '(P_0)$ is positive. The eigenvalue equation satisfied by $\mu (P)$ is

195 $$\begin{aligned} u''-\phi ''=\mu (P)u,\quad -Z\phi ''+\phi =Pu, \end{aligned}$$

where $m(\pm 1/2)=0$ and $\phi$ satisfies periodic boundary conditions. We write $P=P_0+\varepsilon$ for some small $\varepsilon$, and expand *m*, $\phi$, and $\mu$ in $\varepsilon$:

196 $$\begin{aligned} u&=u_0+\varepsilon u_1+O(\varepsilon ^2)\end{aligned}$$

197 $$\begin{aligned} \phi&=\phi _0+\varepsilon \phi _1+O(\varepsilon ^2)\end{aligned}$$

198 $$\begin{aligned} \mu&=\mu _1\varepsilon +O(\varepsilon ^2). \end{aligned}$$

Observe that $\mu _1=\mu '(P_0)$. Solving the zeroth order in $\varepsilon$ equation, we find $u_0$ and $\phi _0$ up to a multiplicative constant:

199 $$\begin{aligned}  &   u_0=\frac{x}{P_0-1}-\frac{1}{2}\frac{P_0}{P_0-1}\csc \left( \frac{\sqrt{P_0-1}}{\sqrt{Z}}\right) \sin \left( \frac{\sqrt{P_0-1} x}{\sqrt{Z}}\right) ,\nonumber \\  &   \quad \phi _0=u_0+x. \end{aligned}$$

Observe that, since $P_0$ and *Z* satisfy (96),

200 $$\begin{aligned} \csc \left( \frac{\sqrt{P_0-1}}{\sqrt{Z}}\right) =\sqrt{\frac{P_0^3-P_0^2+4 Z}{\left( P_0-1\right) P_0^2}}. \end{aligned}$$

In first order, the (195) becomes

201 $$\begin{aligned} u_1''-\phi _1''=\mu _1u_0,\quad -Z\phi _1''+\phi _1=P_0u_1+u_0. \end{aligned}$$

Write $\phi _1=\psi _1+\phi _0/P_0$ where $\psi _1$ solves $-Z\psi _1''+\psi _1=P_0u_1$. Thus, we may write the first order equation as

202 $$\begin{aligned} u_1''-\psi _1''=\mu _1u_0-\frac{1}{Z}u_0+\frac{\phi _0}{P_0Z}. \end{aligned}$$

Since the operator $u_1\mapsto u_1''-\psi _1''$ (which is $S_C(P_0)$) is self adjoint, the right hand side must be orthogonal to the kernel of the operator, which is spanned by $u_0$. Thus, $\mu _1$ solves

203 $$\begin{aligned} \int _{-1/2}^{1/2}\left( \mu _1u_0-\frac{1}{Z}u_0+\frac{\phi _0}{P_0Z}\right) u_0\,{\text {d}}x=0. \end{aligned}$$

Computing the integral and solving for $\mu _1$, we obtain

204 $$\begin{aligned} \mu _1=\frac{3 \left( P_0-1\right) \left( P_0^2-12 Z\right) }{P_0 Z \left( 3 P_0^2-60 Z+2\right) }. \end{aligned}$$

Using Lemma 5.8, we obtain an asymptotic form for $\mu _1$ in large *Z*:

205 $$\begin{aligned} \mu _1=\frac{1}{Z}+O(1/Z^2). \end{aligned}$$

Thus, for sufficiently large *Z*, $\mu '(P_0)=\mu _1>0$.

Lemma 5.2 gives the value of $P_2$. In large *Z*, this expands as

206 $$\begin{aligned} P_2=\frac{\pi ^2}{48}Z+O(1). \end{aligned}$$

Thus, for large *Z*, $P_2>0$. Thus,

207 $$\begin{aligned} \lambda (V)=-\frac{\pi ^2}{24}V^2+O(\frac{V^4}{Z}), \end{aligned}$$

so for large *Z* and small *V*, the largest real part of the eigenvalues of $T_C(V)$ is negative. $\square$

We conclude with a technical lemma used in the proof of Lemma 6.6.

### Lemma 6.8

Suppose $f:[-1/2,1/2]\rightarrow {\mathbb {R}}$ is $C^2$. Let ${\mathcal {B}}=\{v_1,v_2,v_3,\cdots \}$ where $v_n(x)=\sin (n\pi x)$ for *n* odd, and $v_n(x)=\cos (n\pi x)$ for *n* even. Then there exists $C>0$ such that

208 $$\begin{aligned} \sum _{n=1}^\infty |\langle v_m,fv_n\rangle _{L^2}|< C\Vert f\Vert _{L^1}\quad \text {and}\quad \sum _{m=1}^\infty |\langle v_m,fv_n\rangle _{L^2}|< C\Vert f\Vert _{L^1}. \end{aligned}$$

### Proof

Decompose *f* as a Fourier series: $f=\sum _{k=1}^\infty a_kv_k$. Since *f* is $C^2$-smooth, $|a_k|<\Vert f''\Vert _{L^1}/k^2$ Then we can use some product-to-sum trigonometric identities to see that

209 $$\begin{aligned} fv_n=\sum _{k=1}^\infty a_k v_kv_n=\sum _{k=1}^\infty \frac{a_k}{2}(r_{n,k} v_{k+n}+s_{n,k} v_{|k-n|}), \end{aligned}$$

where the the coefficeints $r_{n,k}$ and $s_{n,k}$ are either 1 or $-1$ and are determined by the parities of *n* and *k*. The sign of each coefficient is not important, so we do not endeavor to give them explicitly. Thus,

210 $$\begin{aligned} \sum _{n=1}^\infty |\langle v_m,fv_n\rangle _{L^2}|&=\sum _{n=1}^\infty \left| \sum _{k=1}^\infty \frac{a_k}{2}\langle v_m,r_{n,k} v_{k+n}+s_{n,k}v_{|k-n|}\rangle \right| \end{aligned}$$

211 $$\begin{aligned}&\le \sum _{n=1}^\infty \sum _{k=1}^\infty \frac{|a_k|}{2}(|\langle v_m,v_{k+n}\rangle |+|\langle v_m, v_{|k-n|}\rangle |)\end{aligned}$$

212 $$\begin{aligned}&=\frac{1}{4}\sum _{n=1}^\infty |a_{n+m}|+|a_{|n-m|}|\end{aligned}$$

213 $$\begin{aligned}&\le \frac{3}{4}\sum _{n=1}^\infty |a_n|\end{aligned}$$

214 $$\begin{aligned}&\le \frac{3\Vert f''\Vert _{L^1}}{4}\sum _{n=1}^\infty \frac{1}{n^2}\end{aligned}$$

215 $$\begin{aligned}&=\frac{\pi ^2}{8}\Vert f''\Vert _{L^1}. \end{aligned}$$

Thus the result for the sum over *n* holds. The proof for the sum over *m* is identical. $\square$

**Condition (i):** $T_C$** generates a strongly continuous semigroup, see Appendix C.** Here we show that the linearized operator $T_C$ defined by (155) generates a strongly continuous semigroup. We will make use to of the Hille-Yosida Theorem [41]. We will first prove a supporting proposition.

### Proposition 6.9

There exists $V^*,Z^*,\lambda _0>0$ such that if $|V|<V^*$ and $Z>Z^*$, then all eigenvalues of for all $\lambda >0$ and $u\in \tilde{X}^2_C$

216 $$\begin{aligned} (\lambda -\lambda _0)\left\| u\right\| _{L^2}\le \left\| (\lambda I-T_C)u\right\| _{L^2}. \end{aligned}$$

### Proof

We calculate the norm via the inner product:

$$\begin{aligned} \left\| (\lambda I-T_C)u\right\| _{L^2}^2&=\left\langle (\lambda I-T_C)u,(\lambda I-T_C)u\right\rangle _{L^2}\\&=\lambda ^2\left\| u\right\| _{L^2}^2+\left\| T_Cu\right\| _{L^2}^2-2\lambda \left\langle u,T_Cu\right\rangle _{L^2}. \end{aligned}$$

Observe that

217 $$\begin{aligned}  &   \langle u, T_C u\rangle _{L^2}\le -\Vert u'\Vert _{L^2}^2+\Vert (\phi '(1/2)-\phi )m_{TW}'\Vert _{L^2}^2\nonumber \\  &   \quad +\Vert (V-\phi _{TW})u'\Vert _{L^2}^2+\Vert m_{TW}\phi ''\Vert _{L^2}^2+\Vert u\phi _{TW}''\Vert _{L^2}^2. \end{aligned}$$

There exists $C,V^*>0$ so that $|V|<V^*$ so that (after applying the Poincaré inequality):

218 $$\begin{aligned} \begin{aligned} \Vert (\phi '(1/2)-\phi )m_{TW}'\Vert _{L^2}^2&<C|V|\Vert u\Vert _{L^2}^2\\ \Vert (V-\phi _{TW}')u'\Vert _{L^2}^2&<C|V|\Vert u'\Vert _{L^2}^2\\ \Vert m_{TW}\phi ''\Vert _{L^2}^2&\le C\Vert u\Vert _{L^2}^2\\.\Vert u\phi _{TW}''\Vert _{L^2}^2&\le C|V|\Vert u\Vert _{L^2}^2. \end{aligned} \end{aligned}$$

Assume $V^*$ is sufficiently small that $C|V|<1/2$.

219 $$\begin{aligned} \langle u, T_C u\rangle _{L^2}\le -\frac{1}{2}\Vert u'\Vert _{L^2}^2+(1+C)\Vert u\Vert _{L^2}^2. \end{aligned}$$

Let $\lambda _0=2(1+C)$ so that $\langle u, T_C u\rangle _{L^2}\le (\lambda _0/2)\Vert u\Vert _{L^2}^2$. Thus,

220 $$\begin{aligned} \Vert (\lambda I-T_C)u\Vert _{L^2}^2&\ge \lambda ^2\Vert u\Vert _{L^2}-\lambda \lambda _0\Vert u\Vert _{L^2}^2=(\lambda ^2-\lambda \lambda _0)\Vert u\Vert _{L^2}^2. \end{aligned}$$

If $\lambda >\lambda _0$, then $\lambda ^2-\lambda \lambda _0\ge \lambda ^2-2\lambda \lambda _0+\lambda _0^2=(\lambda -\lambda _0)^2.$ Therefore,

221 $$\begin{aligned} \Vert (\lambda I-T_C)u\Vert _{L^2}\ge (\lambda -\lambda _0)\Vert u\Vert _{L^2}. \end{aligned}$$

$$\square $$

We recall the definition of a closed operator.

### Definition 6.10

Let *X* and *Y* be Banach spaces and let $B:D(B)\subset X\rightarrow Y$ be a linear operator. Then *B* is *closed* if for every sequence $(x_n)$ converging to some $x\in X$ such that $Bx_n$ converges to $y\in Y$, it follows that $x\in D(B)$ and $Bx=y$.

An operator is closed if its resolvent $(\lambda I-B)^{-1}$ exists and is bounded for at least one value of $\lambda \in {\mathbb {C}}$. By Theorem 6.7, the resolvent set of $T_C$ is non-empty, and by Proposition 6.3, the resolvent is compact (and thus bounded) whenever it exists. Therefore, $T_C$ is a closed operator. Thus, we may prove the main result of this section:

### Proposition 6.11

There exists $V^*>0$ such that if $|V|<V^*$, then *A* generates a strongly continuous semigroup.

### Proof

We appeal the the Hille-Yosida Theorem [41], which states that if $T_C:X\rightarrow Y$ is a closed, densely defined operator and if there exists $\lambda _0>0$ such that

222 $$\begin{aligned} \Vert (\lambda I-T_C)^{-n}\Vert _{L^2}\le \frac{1}{(\lambda -\lambda _0)^n}, \end{aligned}$$

then $T_C$ generates a strongly continuous semigroup.

It is clear to see that (222) is satisfied due to Proposition 6.9. Therefore, the hypotheses of the Hille-Yosida theorem are satisfied for sufficiently small $V^*$, so the result holds. $\square$

Since $T_C$ generates a strongly continuous semigroup, the first condition of the Grearhart-Prüss-Griener Theorem is satisfied.

**Condition (iii): the resolvent of** $T_C$** is uniformly bounded, see appendix C.**

Now we prove that the resolvent of $T_C$ is uniformly bounded for complex numbers with positive real part. Then we formally establish linear stability in Theorem 6.13.

### Proposition 6.12

There exist $V^*,Z^*,\Gamma >0$ such that if $0<|V|<V^*$ and $Z>Z^*$, then the resolvent $\Vert (\lambda I-T_C)^{-1}\Vert <\Gamma$ for all $\lambda \in {\mathbb {C}}$ with $\text {Re}\,\lambda >0$.

### Proof

Existence of the resolvent $(\lambda I-T_C)^{-1}$ for all $\lambda$ with $\text {Re}\,\lambda >0$ is established in Theorem 6.7. Assume, to the contrary, that there exists a sequence $(\lambda _k)_{k=1}^\infty \subset {\mathbb {C}}$ such that $\text {Re}\,\lambda _k>0$ for each *k* and

223 $$\begin{aligned} \Vert (\lambda _k I-T_C)^{-1}\Vert _{L^2}>k. \end{aligned}$$

Then for each *k*, there exist $v_k\in \tilde{X}^2_C$ and $w_k\in L^2(-1/2,1/2)$ such that $(\lambda _k I-T_C)v_k=w_k$, $\Vert v_k\Vert _{L^2}=1$, and $\Vert w_k\Vert _{L^2}<1/k$. We shall consider two cases: (i) the sequence $(\lambda _k)$ is bounded, and (ii) $(\lambda _k)$ is unbounded. We will show that in each case, we arrive at a contradiction.

(i) If the sequence $(\lambda _k)$ is bounded, then it has a subsequence also called $(\lambda _k)$ which converges to some $\lambda \in {\mathbb {C}}$ with $\text {Re}\,\lambda \ge 0$. By Theorem 6.7, $\lambda$ is in the resolvent set of $T_C$. Recall the *first resolvent identity* [40] from which we conclude that for each *k*,
  224 $$\begin{aligned} (\lambda I-T_C)^{-1}-(\lambda _k I-T_C)^{-1}=(\lambda -\lambda _k)(\lambda I-T_C)^{-1}(\lambda _k I-T_C)^{-1}.\nonumber \\ \end{aligned}$$
  We calculate:
  $$\begin{aligned} \Vert v_k\Vert _{L^2}&=\Vert (\lambda _kI-T_C)^{-1}w_k\Vert _{L^2}\\&\le \left\| -\left[ (\lambda I-T_C)^{-1}-(\lambda _k I-T_C)^{-1}\right] w_k\right\| _{L^2}+\Vert (\lambda I-T_C)^{-1}w_k\Vert _{L^2}\\&\le \left\| (\lambda _k-\lambda )(\lambda I-T_C)^{-1}(\lambda _k I-T_C)^{-1}w_k\right\| _{L^2}+\Vert w_k\Vert _{L^2}\Vert (\lambda I-T_C)^{-1}\Vert \\&\le |\lambda _k-\lambda |\left\| (\lambda I-T_C)^{-1}v_k\right\| _{L^2}+\Vert w_k\Vert _{L^2}\Vert (\lambda I-T_C)^{-1}\Vert \\&\le \left( |\lambda _k-\lambda |\Vert v_k\Vert _{L^2}+\Vert w_k\Vert _{L^2}\right) \Vert (\lambda I-T_C)^{-1}\Vert _{L^2}. \end{aligned}$$
  Since $|\lambda _k-\lambda _0|,\Vert w_k\Vert _{L^2}\rightarrow 0$ and $\Vert v_k\Vert _{L^2}$ is bounded, we conclude that $\Vert v_k\Vert _{L^2}\rightarrow 0$, a contradiction. Therefore, $(\lambda _k)$ is not bounded.
(ii) If the sequence $(\lambda _k)$ is unbounded, then it has a subsequence also called $(\lambda _k)$ such that $\lambda _k\rightarrow \infty$. There exists corresponding sequences $(v_k)$ and $(w_k)$ such that
  225 $$\begin{aligned} w_k=(\lambda _kI-T_C)v_k,\quad \Vert v_k\Vert _{L^2}=1,\quad \Vert w_k\Vert _{L^2}\le 1/k. \end{aligned}$$
  We calculate the inner product
  226 $$\begin{aligned} \langle w_k,v_k\rangle _{L^2}&=\lambda _k+\Vert v_k'\Vert _{L^2}+\int _{-1/2}^{1/2}(V-\phi _{TW}')v_k'\bar{v}_k\,{\text {d}}x\nonumber \\&+\int _{-1/2}^{1/2}m_{TW}'(\phi _k'(1/2)-\phi _k')\bar{v}_k\,{\text {d}}x-\int _{-1/2}^{1/2}\phi _{TW}''|v_k|^2\,{\text {d}}x\nonumber \\&-\int _{-1/2}^{1/2}m_{TW}\phi _k''\bar{v}_k\,{\text {d}}x \end{aligned}$$
  Since $(v_k)$ is $L^2$-bounded, by Proposition 7.1 in Appendix A, the last three integrals in (226) are uniformly bounded:
  227 $$\begin{aligned} \left| \int _{-1/2}^{1/2}m_{TW}'(\phi _k'(1/2)-\phi _k')\bar{v}_k\,{\text {d}}x-\int _{-1/2}^{1/2}\phi _{TW}''|v_k|^2\,{\text {d}}x-\int _{-1/2}^{1/2}m_{TW}\phi _k''\bar{v}_k\,{\text {d}}x\right| <C\nonumber \\ \end{aligned}$$
  for some $C>0$ independent of *k*.
  Taking the real part of (226), we find using the Cauchy-Schwartz inequality and the Poincaré inequality that
  228 $$\begin{aligned} \text {Re}\,\langle w_k,v_k\rangle \ge \text {Re}\,\lambda _k+\Vert v_k'\Vert _{L^2}-\frac{1}{\pi }\Vert V-\phi _{TW}'\Vert _{L^\infty }\Vert v_k'\Vert _{L^2}^2-C. \end{aligned}$$
  Assuming $V^*$ is sufficiently small that if $|V|<V^*$, then $\Vert V-\phi _{TW}'\Vert _{L^\infty }<\pi$, we conclude that $\text {Re}\,\langle w_k,v_k\rangle \ge \text {Re}\,\lambda _k-C$. On the other hand, $\text {Re}\,\langle w_k,v_k\rangle \le |\langle w_k,v_k\rangle |<1/k$. Since $\text {Re}\,\lambda _k>0$, we conclude that $(\text {Re}\,\lambda _k)$ is bounded. Furthermore, since all terms in (228) have been shown to be bounded except those involving $\Vert v_k'\Vert$, we conclude that $(v_k')$ must be bounded as well.
  Now taking the imaginary part of (226), we find that
  229 $$\begin{aligned} \text {Im}\,\langle w_k,v_k\rangle \ge \text {Im}\,\lambda _k-\frac{1}{\pi }\Vert V-\phi _{TW}'\Vert _{L^\infty }\Vert v_k'\Vert _{L^2}^2-C. \end{aligned}$$
  Once again, all terms in this equation are known to be bounded in *k* except $\text {Im}\,\lambda _k$, so we conclude that $(\text {Im}\,\lambda _k)$ is bounded also, a contradiction.

Since $(\lambda _k)$ can be neither bounded nor unbounded, we conclude that no such sequence $(\lambda _k)$ can exist, and so $(\lambda I-T_C)^{-1}$ is uniformly bounded. That is, there exists $\Gamma >0$ such that

230 $$\begin{aligned} \Vert (\lambda I-T_C)^{-1}\Vert <\Gamma . \end{aligned}$$

$$\square $$

Now that we have in place all the results proving the conditions of the GPG theorem, we may apply it to prove linear stability.

### Theorem 6.13

There exist $V^*,Z^*,\Gamma ,\sigma >0$ such that if $|V|<V^*$ and $Z>Z^*$ then $T_C$ generates a strongly continuous semigroup $\{S(t):t\ge 0\}$ satisfying

231 $$\begin{aligned} \Vert S(t)\Vert <\Gamma e^{-\sigma t}. \end{aligned}$$

### Proof

We need to satisfy the three hypotheses of the GPG theorem 9.1, see Appendix C. We have checked that indeed:

- (i) is satisfied for sufficiently small $V^*$ due to Proposition 6.11
- (ii) is satisfied for sufficiently small $V^*$ and sufficiently large $Z^*$ due to Theorem 6.7.
- (iii) is satisfied due to Proposition 6.12.

Thus, the desired result holds. $\square$

The last key piece of the proof of Theorem 6.1 is that the linear part of $F_C$ dominates the nonlinear part near $m_{TW}$, which is proved by the following lemma:

### Lemma 6.14

Let $T,\delta >0$ and let *u* be a solution to

232 $$\begin{aligned} {\left\{ \begin{array}{ll} \partial _tu=T_Cu+\Psi (u) &  -1/2<x<1/2,\;0<t<T\\ u'=0 &  x=\pm 1/2,\;t>0. \end{array}\right. } \end{aligned}$$

There exist $V^*,U^*>0$ such that if $\Vert u'(0,\cdot )\Vert _{L^2}<\delta$, $|V|<V^*$, and $\Vert u(\cdot ,t)\Vert _{L^2}<U^*$ for all $0\le t\le T$, then

233 $$\begin{aligned} \Vert u'(\cdot ,t)\Vert _{L^2}\le \delta \end{aligned}$$

for all $$0\le t\le T$$.

### Proof

Write the evolution equation (46) as

234 $$\begin{aligned} \partial _t u-u''=Bu+\Psi (m). \end{aligned}$$

Where *B* is defined by

235 $$\begin{aligned} Bu= &   (\phi '(1/2)-\phi ')m_{TW}'+(V-\phi _{TW}')u'-m_{TW}\phi ''\nonumber \\  &   -u\phi _{TW}'',\quad {\left\{ \begin{array}{ll}-Z\phi ''+\phi =Pu\\ \phi (-1/2)=\phi (1/2)\\ \phi '(-1/2)=\phi '(1/2).\end{array}\right. } \end{aligned}$$

Now square both sides and integrate to obtain

236 $$\begin{aligned} \Vert Bu+\Psi (u)\Vert _{L^2}^2&=\int _{-1/2}^{1/2}\Psi ^2(u)\,{\text {d}}x \end{aligned}$$

237 $$\begin{aligned}&=\int _{-1/2}^{1/2} (\partial _t u)^2-2(\partial _t u)u''+(u'')^2\,{\text {d}}x\end{aligned}$$

238 $$\begin{aligned}&=\Vert \partial _t u\Vert _{L^2}^2+2\int _{-1/2}^{1/2}(\partial _t u')u'\,{\text {d}}x+\Vert m''\Vert _{L^2}^2\end{aligned}$$

239 $$\begin{aligned}&=\Vert \partial _t u\Vert _{L^2}^2+\frac{\text {d}}{{\text {d}}t}\Vert u'\Vert _{L^2}^2+\Vert u''\Vert _{L^2}^2. \end{aligned}$$

Thus,

240 $$\begin{aligned} \frac{\text {d}}{{\text {d}}t}\Vert u'\Vert ^2_{L^2}&\le \Vert Bu+\Psi (u)\Vert _{L^2}^2-\Vert u''\Vert _{L^2}^2\end{aligned}$$

241 $$\begin{aligned}&\le 2\Vert Bu\Vert _{L^2}^2+2\Vert \Psi (u)\Vert _{L^2}^2-\Vert u''\Vert _{L^2}^2. \end{aligned}$$

From Lemma 4.8, there exists $C_1$ independent of *u*, *V*, and *Z* such that

242 $$\begin{aligned} \Vert \Psi (u)\Vert _{L^2}\le C_1\Vert u\Vert _{L^2}\Vert u\Vert _{H^1}. \end{aligned}$$

Observe that due to 7.1, if $|V|<V^*$ is small enough, there exist $C_2$, $C_3$ depending only on *Z* such that

243 $$\begin{aligned} \Vert Bu\Vert _{L^2}\le C_2V^*\Vert u'\Vert _{L^2}+C_3\Vert u\Vert _{L^2}. \end{aligned}$$

Since $\int _{-1/2}^{1/2}u\,{\text {d}}x=0$ and $u'(\pm 1/2,t)=0$, we may apply the Poincaré inequality to both *u* and $u'$ with a Poincaré constant of $\pi$:

244 $$\begin{aligned} \pi \Vert u\Vert _{L^2}\le \Vert u'\Vert _{L^2}\quad \text {and}\quad \pi \Vert u'\Vert _{L^2}\le \Vert u''\Vert _{L^2}. \end{aligned}$$

Thus,

245 $$\begin{aligned} \frac{\text {d}}{{\text {d}}t}\Vert u'\Vert ^2_{L^2}&\le 2C_1^2\Vert u\Vert _{L^2}^2\Vert u\Vert _{H^1}^2+4C_2^2(V^*)^2\Vert u'\Vert _{L^2}^2+4C_3^2\Vert u\Vert _{L^2}^2-\Vert u''\Vert _{L^2}^2\end{aligned}$$

246 $$\begin{aligned}&\le 2C_1^2\left( 1+\frac{1}{\pi }\right) ^2\Vert u\Vert _{L^2}^2\Vert u'\Vert _{L^2}+4C_2^2(V^*)^2\Vert u'\Vert _{L^2}^2+4C_3^2\Vert u\Vert _{L^2}^2-\Vert u''\Vert _{L^2}^2\end{aligned}$$

247 $$\begin{aligned}&\le -\left( \pi ^2-4C_1^2\Vert u\Vert _{L^2}^2-4C_2^2(V^*)^2\right) \Vert u'\Vert _{L^2}^2+4C_3^2\Vert u\Vert _{L^2}^2. \end{aligned}$$

Without loss of generality, we may assume that

248 $$\begin{aligned} V^*\le \frac{\pi }{4C_2}\quad \text {and}\quad U^*\le \frac{\pi }{4C_1}. \end{aligned}$$

Then, if $\Vert u\Vert _{L^2}\le U^*$ for all $0<t<T$,

249 $$\begin{aligned} \frac{\text {d}}{{\text {d}}t}\Vert u'\Vert ^2_{L^2}\le -R_1\Vert u'\Vert _{L^2}^2+R_2 \end{aligned}$$

where

250 $$\begin{aligned} R_1=\pi ^2-4C_1^2(U^*)^2-4C_2^2(V^*)^2\ge \frac{\pi ^2}{2}\quad \text {and}\quad R_2=4C_3^2(U^*)^2. \end{aligned}$$

We now introduce a new variable:

251 $$\begin{aligned} q(t)=\Vert u'(\cdot ,t)\Vert _{L^2}^2-\frac{R_2}{R_1}. \end{aligned}$$

Then *q* satisfies $q'\le -R_1q.$ By Grönwall’s inequality,

252 $$\begin{aligned} q(t)\le q(0)e^{-R_1t}. \end{aligned}$$

We conclude that if $q(0)<0$, then $q(t)<0$ for all $t>0$. Thus, if $|V|<V^*$ and $\Vert u\Vert _{L^2}\le U^*$, and if

253 $$\begin{aligned} \Vert u'(\cdot ,0)\Vert _{L^2}<\sqrt{\frac{R_2}{R_1}},\quad \text {then}\quad \Vert u'(\cdot ,t)\Vert _{L^2}<\sqrt{\frac{R_2}{R_1}} \end{aligned}$$

for all $t>0$. Letting $U^*$ be sufficiently small that

254 $$\begin{aligned} \sqrt{\frac{R_2}{R_1}}\le \frac{\sqrt{8}}{\pi }C_3 U^*<\delta , \end{aligned}$$

the desired result holds. $\square$

With Lemma 6.14 in place, we may duplicate the proof of Theorem 4.1 in order to prove the nonlinear stability of traveling waves via Theorem 6.1.

## Data Availability

This manuscript does not report any datasets or generated data. All results are theoretical, and no data were used or produced in this study.

## Appendix A

Here we show the Proposition which controls the solution $\phi$ to (34), (37) and (38).

### Proposition 7.1

Let $u\in L^0([-1/2,1/2])$. Then there exists a unique solution $\phi \in W^{2,p}(-1/2,1/2)$ for any $1\le p\le \infty$ satisfying $-Z\phi ''+\phi =Pu$ with periodic boundary conditions on $(-1/2,1/2)$. Moreover, $\phi$ satisfies the following for any $1\le p\le \infty$:

- $$\Vert \phi \Vert _{L^p}\le P\Vert u\Vert _{L^p}$$,
- $$\Vert \phi '\Vert _{L^\infty }\le \frac{P}{2Z}\Vert u\Vert _{L^2}$$,
- $$\Vert \phi ''\Vert _{L^p}\le \frac{2P}{Z}\Vert u\Vert _{L^p}$$.

### Proof

The solution $\phi$ can be calculated explicitly using a Green’s function:

255 $$\begin{aligned}  &   \phi (x)=\frac{P}{2\sqrt{Z}\sinh \left( \frac{1}{2\sqrt{Z}}\right) }\int _{-1/2}^{1/2}G(x,y)u(y)\,dy,\nonumber \\  &   \quad G(x,y)={\left\{ \begin{array}{ll} \cosh \left( \frac{1/2+(y-x)}{\sqrt{Z}}\right) &  y<x\\ \cosh \left( \frac{1/2+(x-y)}{\sqrt{Z}}\right) &  y>x. \end{array}\right. }. \end{aligned}$$

By Young’s Integral inequality [85], $\Vert \phi \Vert _{L^p}\le C\Vert u\Vert _{L^p}$ where

256 $$\begin{aligned}  &   C=\sup _{|x|\le 1/2}\frac{P}{2\sqrt{Z}\sinh \left( \frac{1}{2\sqrt{Z}}\right) }\int _{-1/2}^{1/2}|G(x,y)|\,dy\nonumber \\  &   \quad =\sup _{|y|\le 1/2}\frac{P}{2\sqrt{Z}\sinh \left( \frac{1}{2\sqrt{Z}}\right) }\int _{-1/2}^{1/2}|G(x,y)|\,{\text {d}}x. \end{aligned}$$

We calculate

257 $$\begin{aligned} \frac{P}{2\sqrt{Z}\sinh \left( \frac{1}{2\sqrt{Z}}\right) }&\int _{-1/2}^{1/2}|G(x,y)|\,dy \end{aligned}$$

258 $$\begin{aligned}&=\frac{P}{2\sqrt{Z}\sinh \left( \frac{1}{2\sqrt{Z}}\right) }\left( \int _{-1/2}^x\cosh \left( \frac{1/2+y-x}{\sqrt{Z}}\right) \,dy\right. \nonumber \\&\quad \left. +\int _x^{1/2}\cosh \left( \frac{1/2+x-y}{\sqrt{Z}}\right) \,dy\right) \end{aligned}$$

259 $$\begin{aligned}&=\frac{P}{2\sinh \left( \frac{1}{2\sqrt{Z}}\right) }\left( \sinh \left( \frac{1}{2\sqrt{Z}}\right) +\sinh \left( \frac{x}{\sqrt{Z}}\right) \right. \nonumber \\&\quad \left. -\sinh \left( \frac{x}{\sqrt{Z}}\right) +\sinh \left( \frac{1}{2\sqrt{Z}}\right) \right) \end{aligned}$$

260 $$\begin{aligned}&=P. \end{aligned}$$

We conclude that $\Vert \phi \Vert _{L^p}\le P\Vert u\Vert _{L^p}$. Next, since *G* is continuous and differentiable in *x* except where $x=y$,

261 $$\begin{aligned} \phi '(x)= \frac{P}{2\sqrt{Z}\sinh \left( \frac{1}{2\sqrt{Z}}\right) }\int _{-1/2}^{1/2}\frac{d}{{\text {d}}x}G(x,y)u(y)dy. \end{aligned}$$

Therefore, using Hölder’s inequality for *p* and its Hölder conjugate *q*,

262 $$\begin{aligned} \Vert \phi '\Vert _{L^\infty }\le \frac{P}{2\sqrt{Z}\sinh \left( \frac{1}{2\sqrt{Z}}\right) }\Vert u\Vert _{L^p}\sup _{|x|\le 1/2}\left\| \frac{d}{{\text {d}}x}G(x,\cdot )\right\| _{L^q}. \end{aligned}$$

Since $|\frac{d}{{\text {d}}x}G(x,y)|\le \frac{1}{\sqrt{Z}}\sinh \left( \frac{1}{2\sqrt{Z}}\right)$, we conclude that $\Vert \phi '\Vert _{L^\infty }\le \frac{P}{2Z}\Vert u\Vert _{L^p}.$ Finally, since $\phi ''=-\frac{P}{Z}u+\frac{1}{Z}\phi$, we have

263 $$\begin{aligned} \Vert \phi ''\Vert _{L^p}\le \frac{P}{Z}\Vert u\Vert _{L^p}+\frac{1}{Z}\Vert \phi \Vert _{L^p}\le \frac{2P}{Z}\Vert u\Vert _{L^p}. \end{aligned}$$

$$\square $$

## Appendix B

Here we formulate for convenience the Crandall-Rabinowitz (CR) theorem [25].

### Theorem 8.1

Let *X* and *Y* be Banach spaces, and let ${\mathcal {F}}:X\times {\mathbb {R}}\rightarrow Y$ be an operator with the following properties:

- $${\mathcal {F}}(0,t)=0$$ for all *t*.
- $D_x{\mathcal {F}}$, $D_t{\mathcal {F}}$, and $D_{xt}{\mathcal {F}}$ exist and are continuous.
- The dimension of the null space and co-dimension of the range of $D_x{\mathcal {F}}(0,0)$ are both 1.
- If $x_0\ne 0$ is in the null space of $D_x{\mathcal {F}}(0,0)$, then $D_{xt}{\mathcal {F}}(0,0)x_0$ is not in the range of $D_x{\mathcal {F}}(0,0)$.

Then there exists a neighborhood $U\subset X\times {\mathbb {R}}$ of (0, 0), $\varepsilon >0$ and functions $\sigma :(-\varepsilon ,\varepsilon )\rightarrow X$ and $s:(-\varepsilon ,\varepsilon )\rightarrow {\mathbb {R}}$ with $\sigma \not \equiv 0$ such that $\sigma (0)=0$, $s(0)=0$, and

264 $$\begin{aligned} {\mathcal {F}}^{-1}(0)\cap U=\left( \{(0,t):t\in {\mathbb {R}}\}\cup \{(\sigma (\alpha ),s(\alpha )):|\alpha |<\varepsilon \}\right) \cap U. \end{aligned}$$

Moreover, if ${\mathcal {F}}_{xx}$ exists and is continuous, then $\sigma$ is continuously differentiable and $\sigma '(0)$ spans the null space of $D_x{\mathcal {F}}(0,0)$.

In the main text we proceed by checking systematically these four properties for the operator (134).

## Appendix C

Here we formulate the the Gearhart-Prüss-Greiner (GPG) theorem [41].

### Theorem 9.1

Let *X* be a Hilbert space, and let $L:D(L)\rightarrow X$ be a linear operator, where the domain *D*(*L*) of *L* is a dense subspace of *X*. If the following conditions hold

(i) the semigroup $(S(t))_{t\ge 0}$ generated by *L* is strongly continuous,
(ii) The resolvent set of *L* contains $\{z\in {\mathbb {C}}:\text {Re}\,z>0\}$, and
(iii) The resolvent $(\lambda I-L)^{-1}$ is uniformly bounded on the above set, i.e.,
  265 $$\begin{aligned} \sup _{\text {Re}\,\lambda >0}\Vert (\lambda I-L)^{-1}\Vert _X<\infty , \end{aligned}$$

then there exists $\Gamma ,\sigma >0$ such that

- For each $\lambda$ in the spectrum $\sigma (S(t))$, $|\lambda |<e^{-\sigma t}$, and
- For each $$t\ge 0$$, $$\Vert S(t)\Vert _X\le \Gamma e^{-\sigma t}$$.

In the main text we proceed by checking systematically these three conditions for the operator (155).

## Footnotes

1 It should be noted that in some specific NSA problems, eigenvectors do span the whole domain and in those cases the absence of negative eigenvalues may be sufficient for stability, e.g. [62].
